# Type 2 Diabetes Promotes the Microglial Pyroptosis by Activating NLRP3 Inflammasome to Impede Remyelination After Spinal Cord Injury

**DOI:** 10.34133/research.1237

**Published:** 2026-04-14

**Authors:** Jingyu Xu, Li Fang, Tao Wei, Shufang Cai, Ran Chen, Bingbin Wang, Saiqun Nie, Yueqi Wu, Fuyi Yan, Jiaqin Shao, Wenjun Wu, Ying Zhan, Ke Xu, Jian Xiao, Yanqing Wu

**Affiliations:** ^1^ The Institute of Life Sciences, Engineering Laboratory of Zhejiang Province for Pharmaceutical Development of Growth Factors, Wenzhou University, Wenzhou, Zhejiang 325035, China.; ^2^ Department of Endocrinology, The First Affiliated Hospital and School of Pharmaceutical Sciences, Wenzhou Medical University, Wenzhou, Zhejiang 325035, China.; ^3^ The College of Life Sciences, Northwest University, Xi’an 710127, China.; ^4^ Department of Hand Surgery, Ningbo No. 6 Hospital, Ningbo 315043, China.

## Abstract

Diabetes hinders nerve recovery after spinal cord injury (SCI). The complex pathological factors of diabetes increase the difficulty of treating diabetes combined with SCI. Maintaining normal microglial function is essential for SCI recovery. However, it is unclear whether diabetes hinders nerve recovery after SCI by influencing normal microglial function. This study explored the role and regulatory mechanism of diabetes in microglial function during SCI recovery. We constructed a type 2 diabetes (T2D) combined with SCI mouse model and confirmed that T2D hinders nerve repair after SCI. T2D blocked phagocytizing function of microglia in SCI mice, which results in increased myelin debris accumulation and poor remyelination. A mechanistic study demonstrated that T2D triggers activation of NLRP3 inflammasome by activating the RAGE–ROS–TXNIP axis and then induces excessive microglial pyroptosis, which consequently leads to considerable loss of microglia after SCI. Verapamil (VRP; a TXNIP inhibitor) treatment confirmed that TXNIP is necessary for NLRP3 inflammasome activation. Conditional microglial *Caspase-1* gene knockout (KO) mice also confirmed that excessive microglial pyroptosis is an important inducing event for more severe nerve damage in T2D combined with SCI mice. Moreover, this T2D effect on increased microglial pyroptosis was also effectively reversed by *N*-acetyl-l-cysteine (NAC; an antioxidant) and *N*-benzyl-4-chloro-*N*-cyclohexylbenzamide (FPS-ZM1; a RAGE inhibitor). In conclusion, this study revealed that T2D induces increased microglial pyroptosis by activating the RAGE–ROS–NLRP3 axis, and then blocks remyelination after SCI, which strongly suggests that microglial pyroptosis may be the key target for treating T2D combined with SCI.

## Introduction

Spinal cord injury (SCI), a serious neurological trauma, can cause severe motor dysfunction in areas below the level of injury [1–4]. After SCI, it rapidly triggers blood–spinal cord barrier (BSCB) destruction, nerve cell death, and demyelination, followed with series of secondary injuries. Among them, the intense inflammatory response is an important secondary injury that limits SCI recovery [5]. Additionally, the accumulation of myelin debris surrounding lesion sites further increases the difficulty of nerve repair after SCI [2,6]. Diabetes is closely related to nerve repair after SCI, and SCI patients are more prone to develop diabetes because of a lack of exercise [7]. In turn, chronic hyperglycemia is more prone to cause epidural abscess and spinal cord infarction and leads to nerve injury [8,9]. We, and others, have reported that diabetes impedes SCI recovery [3,10,11]. Currently, the clinical therapeutic methods for treating SCI are limited, and the curative effects are imprecise. The complicated pathological factors of diabetes make the clinical treatment of diabetes combined with SCI more difficult [12]. Therefore, an in-depth exploration of the regulatory mechanism responsible for diabetes hindering SCI recovery is urgently needed.

The intense inflammatory response is an important pathological feature after SCI, which severely hinders the neural repair process after SCI. After SCI, a typical continuous inflammatory cascade occurs and runs through the whole SCI process, which facilitates the degeneration and necrosis of neurons and glial cells in the injured area. The normal function of glial cells is highly important for neural repair after SCI. Among them, microglia exert a crucial regulatory role in chronic neuroinflammation [13,14]. Specifically, microglia are rapidly activated and transformed into the M1 type after SCI, which results in the formation of a cytokine network for pro- or anti-inflammatory responses that peaks within 3 to 7 d [15]. Microglial cell-dependent inflammation expands the scope of inflammation and exacerbates the secondary injury after SCI [16,17]. More importantly, microglia also constitute the innate immune system together with phagocytes differentiated from blood-derived monocytes, which are responsible for timely removal of myelin debris and consequently promote remyelination. Therefore, normal microglial function plays a key role in remyelination and axonal regeneration during SCI recovery. Diabetic condition has been demonstrated to greatly induce excessive inflammation in the microglia of SCI patients and mice [18]. Thus, we propose that diabetes may hinder SCI recovery by disturbing normal microglial function.

Pyroptosis is a newly discovered form of programmed cell death. During pyroptosis, pattern recognition receptors in the inflammasome recognize specific molecular patterns, and then recruit and activate the adaptor apoptosis-associated speck-like protein containing a caspase-recruitment domain (ASC). Activated ASC promotes the maturation of cysteinyl aspartate specific proteinase-1 (Caspase-1) through caspase recruitment domain (CARD)-CARD interactions and then promotes N domain-gasdermin D (N-GSDMD) formation. Finally, mature interleukin-18 (IL-18) and interleukin-1β (IL-1β) are released and consequently result in swelling and rupture of cell [19,20]. Pyroptosis is widely involved in the regulatory process of nerve repair after SCI. It is reported that inhibiting the voltage-gated proton channel of microglia alleviates NOD-like receptor protein 3 (NLRP3) inflammasome-associated neuronal pyroptosis by suppressing reactive oxygen species (ROS) generation, subsequently promoting SCI recovery [21]. After SCI, Toll-like receptor 4 (TLR4)/MyD88 signaling activation also significantly promotes pyroptosis of macrophages/microglia [22]. More importantly, diabetes accompanied by a chronic diffuse systemic inflammatory response is also closely related to excessive pyroptosis in the body [23,24]. Thus, we hypothesized that pyroptosis may be a critical event in type 2 diabetes (T2D) disturbing normal function of microglia during SCI recovery.

Here, a T2D combined with SCI mouse model was established to explore the role and regulatory mechanism of T2D in microglial function after SCI. The results revealed that T2D greatly induces excessive microglial pyroptosis and disturbs normal microglial function by activating the receptor of advanced glycation end products (RAGE)–ROS–NLRP3 axis, consequently blocking remyelination of the spinal cord and hindering SCI recovery. These results strongly support that microglial pyroptosis may be the key target by which T2D hinders SCI treatment.

## Results

### T2D hindered locomotor functional recovery in SCI mice

**Fig. 1.**
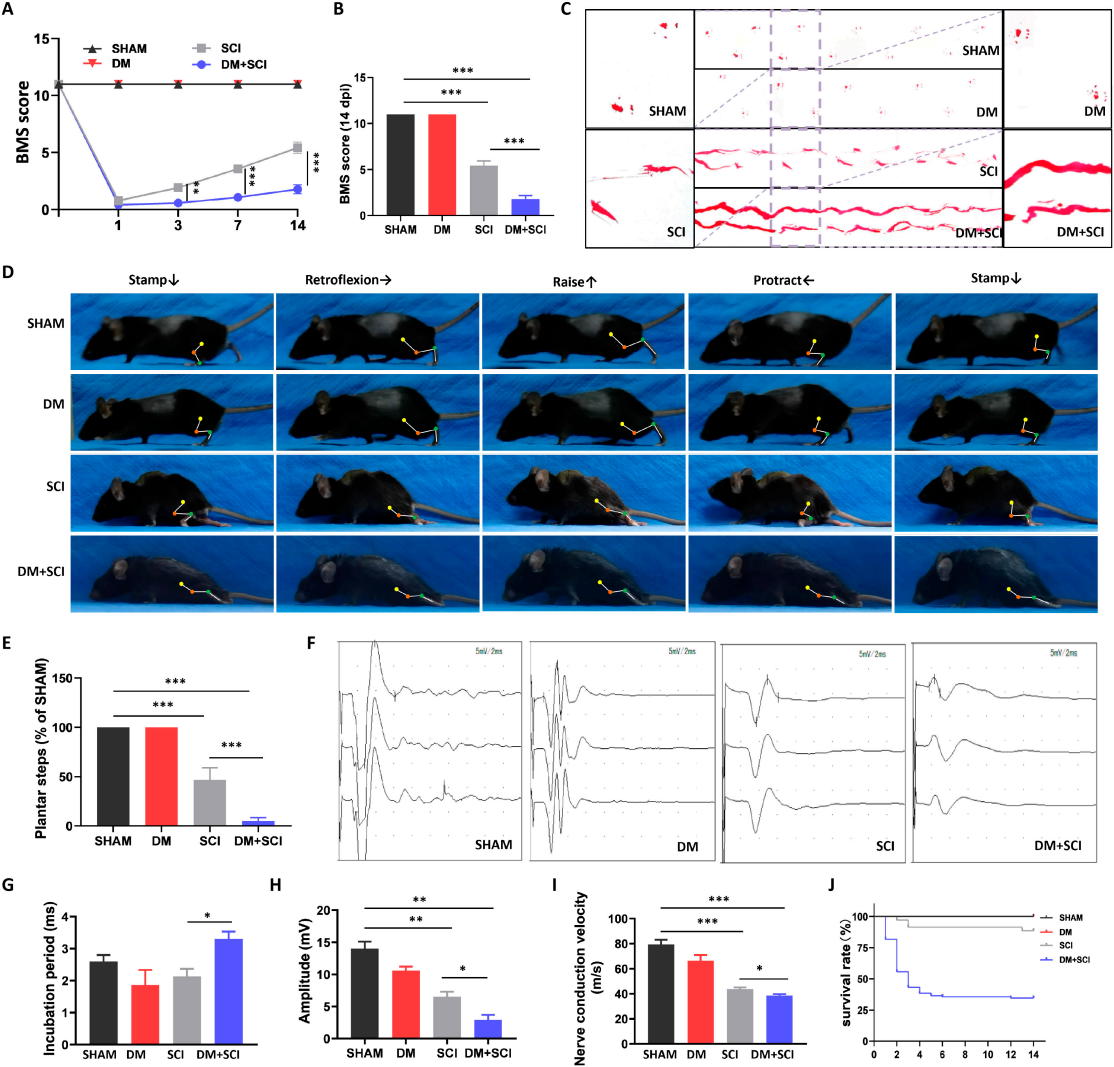
T2D hindered locomotor functional recovery in SCI mice. (A) BMS scores of the hind limbs of the mice on days 1, 3, 7, and 14 post-SCI. (B) Statistical analysis of the BMS score on day 14 post-SCI (*n* = 12). (C) Footprints of the mice on day 14 post-SCI (*n* = 6). (D and E) Motion dynamic images of the mice and statistical analysis of the plantar landing time of the mice on day 14 post-SCI (*n* = 6). The ankle, knee, and hip joints are marked with lines and dots, and the direction of foot movement is indicated by arrows. The electrophysiological results (F) and the analysis of incubation period (G), amplitude (H), and nerve conduction velocity (I) of electrical signals in hind limb of mice on day 14 post-SCI (*n* = 3). (J) Survival rate of the mice within 14 d after SCI (*n* = 12). *P < 0.05, **P < 0.01, ***P < 0.001.

Diabetes aggravated the nerve damage in SCI mice. After SCI, the basso mouse scale (BMS) scores of both the mice in the SCI group and the diabetes mellitus (DM) + SCI group sharply declined to a score nearly 0. Subsequently, the BMS score of the mice in the SCI group was gradually enhanced with a score of 3.5 on day 7 post-SCI and 5.5 on day 14 post-SCI (Fig. 1A and B). Compared with SCI mice, DM + SCI model mice presented worse locomotor function with a much lower BMS score of 1.1 on day 7 post-SCI and 1.8 on day 14 post-SCI (Fig. 1A and B). The footprints and motion dynamic images of the mice also demonstrated that both hind limbs of the SCI mice could be lifted slightly and turn instep with a break point shape in the footprints (Fig. 1C to E). However, the hind limbs of the mice in the DM + SCI group were much more difficult to lift, the instep of hind limbs faced down, the mice were unable to turn and lift, and the footprints also had persistent dragging phenomena (Fig. 1C to E). The electrophysiology results also presented a significantly prolonged incubation period, decreased amplitude, and slow nerve conduction velocity of hind limb in mice from the DM + SCI group (Fig. 1F to I). More importantly, we also tracked the survival rate of the mice within 14 d after SCI. T2D led to the much lower survival rate of the SCI mice (35%) than that in the SCI group (80%) (Fig. 1J). The mortality of DM + SCI mice substantially increased within 3 d after SCI (Fig. 1J). More importantly, there were no potential effects of streptozotocin (STZ) toxicity independent of hyperglycemia on locomotor function of mice (Fig. S1). These results indicate that T2D significantly aggravates SCI and even promotes the mortality of SCI mice.

### T2D impeded microglial function and nerve repair in SCI mice

**Fig. 2.**
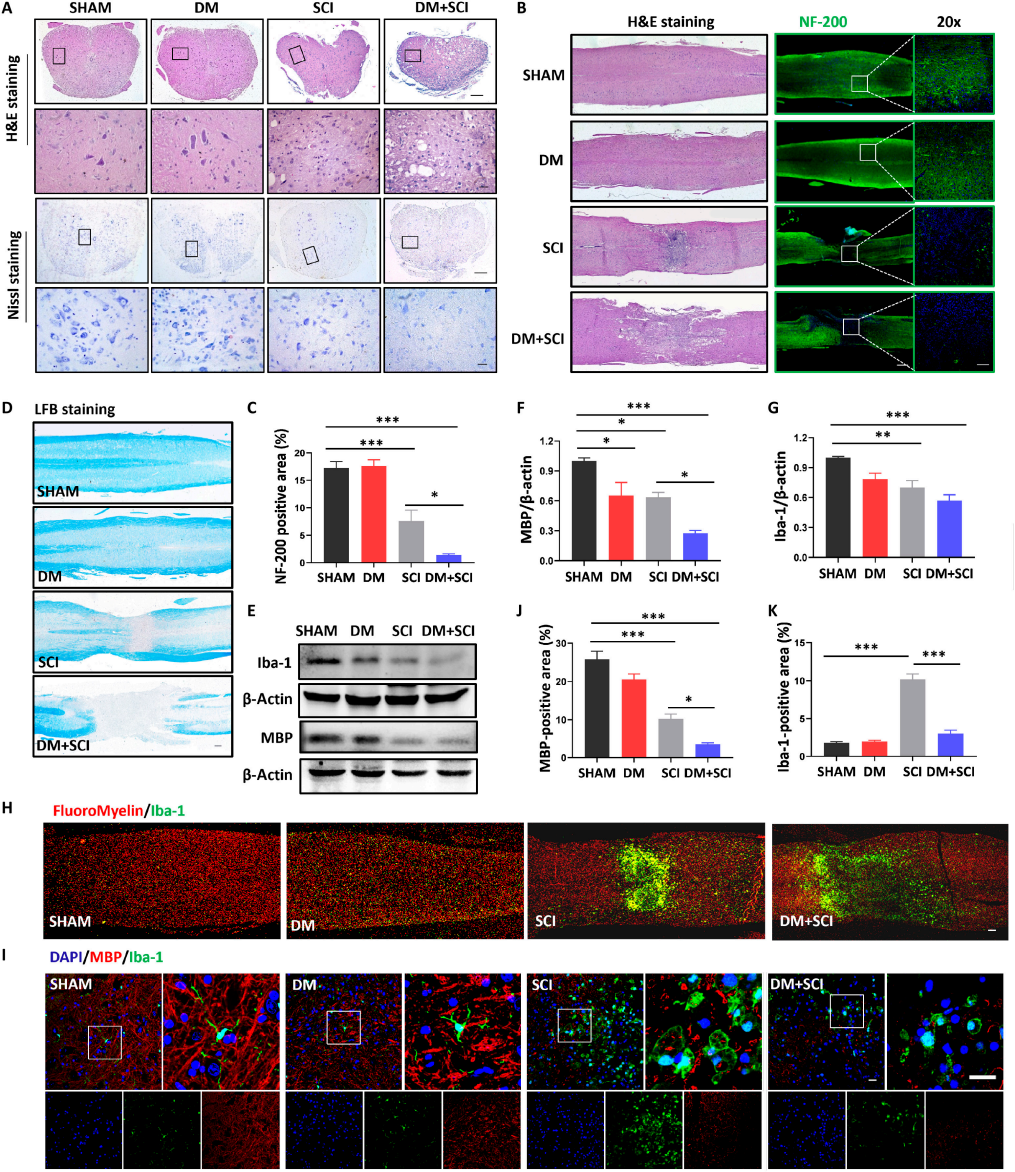
T2D impeded microglial function and nerve repair in SCI mice. (A) H&E staining (*n* = 5) and Nissl staining (*n* = 3) of transverse sections of the spinal cord on day 14 post-SCI. Scale bars, 25 and 400 μm. (B) H&E staining (*n* = 5) and NF-200 (green) staining (*n* = 3) of longitudinal sections of the spinal cord on day 14 post-SCI. Scale bar, 100 μm. (C) Statistical analysis of NF-200 staining in (B). (D) LFB staining of longitudinal sections of the spinal cord on day 14 post-SCI (*n* = 3). (E to G) WB results and statistical analysis of Iba-1 and MBP in the spinal cord on day 5 post-SCI (*n* = 3). (H) Costaining of FluoroMyelin (red) and Iba-1 (green) in the spinal cord on day 14 post-SCI. Scale bar, 100 μm. *n* = 3. (I to K) Costaining of MBP (red) and Iba-1 (green) in the spinal cord on day 5 post-SCI. Scale bar, 20 μm. *n* = 3. *P < 0.05, **P < 0.01, ***P < 0.001.

We next detected the effect of T2D on axon regeneration and remyelination of the spinal cord after SCI. Although the spinal cords in the SCI group presented partial loss of Nissl bodies, the morphology of spinal cords remained relatively unbroken (Fig. 2A and B). As shown in Fig. 2A and B, the spinal cords in the DM + SCI group presented a larger injured area with a much greater void area than those in the SCI group. Nissl staining also revealed that T2D markedly induced the loss of Nissl bodies in the spinal cord of SCI mice (Fig. 2A). Neurofilament-200 (NF-200) staining further revealed that neurofilaments gradually extended to the injured site in the mice of the SCI group and even extended to the epicenter (Fig. 2B and C). However, the neurofilament cavity area in the DM + SCI group was larger than that in the SCI group, with many fewer neurofilaments at the epicenter (Fig. 2B and C). In addition, the spinal cords in the DM + SCI group displayed severe demyelination at the damage epicenter, with a larger area of nonpositive Luxol fast blue (LFB) signals and a reduced myelin basic protein (MBP) expression level (Fig. 2D to F).

Microglia exert a critical role in phagocytosing the myelin fragments and promoting remyelination during SCI recovery. As shown in Fig. 2E and G, T2D suppressed Iba-1 (a microglial marker) expression in the spinal cord of SCI mice. We also assessed the size of demyelinating lesion with FluoroMyelin and used Iba-1 to label microglia. We observed a greater demyelinating lesion volume in DM + SCI mice, and a decrease in Iba-1^+^ cell density, indicating the poor remyelination in DM + SCI mice compared with SCI mice (Fig. 2H). Moreover, costaining for MBP and Iba-1 further revealed that the morphology of microglia in the SHAM and DM groups were both in a quiescent state and exhibited a branched morphology (Fig. 2I). After SCI, microglia aggregated at the injured site and formed vesicles, and then, the vesicles became larger and phagocytosed myelin debris (Fig. 2I to K). T2D not only significantly induced the loss of microglia but also led to a smaller cell body and poor ability to phagocytose myelin debris even though the microglia also appeared vesicles (Fig. 2I to K). These results indicate that the loss of microglia and their poor phagocytizing function may severely restrict remyelination and nerve repair in T2D combined with SCI mice.

### T2D altered the transcription of genes in the spinal cord of SCI mice

**Fig. 3.**
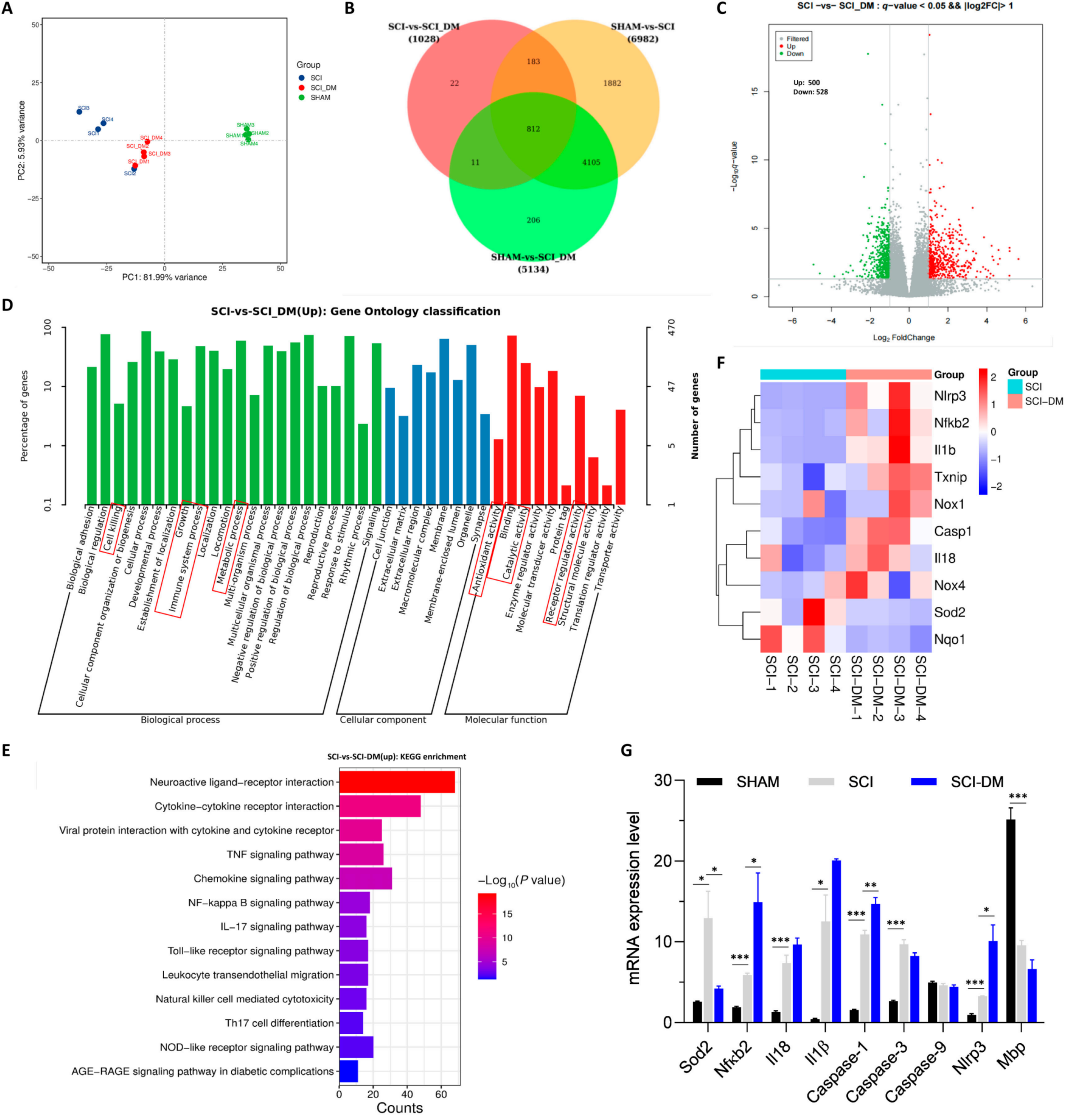
T2D significantly altered the transcription of genes in the spinal cord in SCI mice. (A) PCA. (B) Venn diagram. (C) Volcano map. (D) GO enrichment analysis. (E) KEGG enrichment analysis. (F) Heatmap showing the DEGs related to pyroptosis and oxidative stress. (G) Statistical analysis of the mRNA levels of *Sod 2*, *Nfkb2*, *Caspase-1*, *Nlrp3*, *Il18*, *Il1β*, *Caspase-3*, *Caspase-9*, and *Mbp* in the spinal cord from different groups. *n* = 4, *P < 0.05, **P < 0.01, ***P < 0.001.

To further explore the regulatory mechanism by which diabetes hinders microglial function after SCI, we performed the RNA sequencing (RNA-Seq) analysis of the spinal cord. The principal components analysis (PCA) and Venn diagram analysis revealed a total of 1,028 differentially expressed genes (DEGs) between the SCI and SCI-DM groups (Fig. 3A to C). There are 500 up-regulated genes and 528 down-regulated genes (Fig. 3C). Gene Ontology (GO) enrichment analysis further revealed that biological processes in regard to the immune system process, cell killing, metabolic process, and growth were significantly enriched in the SCI-DM group (Fig. 3D). In terms of molecular function, binding, antioxidant activity, receptor regulator activity, and catalytic activity were prominently enriched in the SCI-DM group (Fig. 3D). Kyoto Encyclopedia of Genes and Genomes (KEGG) pathway enrichment analysis further indicated the obvious enrichment in the pathways associated with natural killer cell-mediated cytotoxicity, tumor necrosis factor (TNF) signaling pathway, TLR signaling pathway, nuclear factor κB (NF-κB) signaling pathway, chemokine signaling pathway, and cytokine–cytokine receptor interaction in the SCI-DM group (Fig. 3E). Pyroptosis is a new type of programmed cell death closely related to inflammatory responses. Thus, we further analyzed the expression of genes related to pyroptosis. The heatmap of DEGs related to pyroptosis, inflammation, and oxidative stress showed that compared with the SCI group, there were increased expression of pyroptosis (*Nlrp3* and *Casp-1*), pro-inflammation (*Nfκb2*, *Il18*, and *Il1β*), and prooxidant (*Nox4*, *Nox1*, and *Txnip*)-related genes and decreased expression of antioxidant-related genes (*Sod2* and *Nqo1*) in the spinal cord from the SCI-DM group (Fig. 3F). Compared with the SHAM group, SCI significantly increased the mRNA level of *Sod2*, *Nfκb2*, *Caspase-1*, *Nlrp3*, *Il18*, *Il1β*, and *Caspase-3* and decreased the mRNA level of *Mbp* in the spinal cord (Fig. 3G). More interestingly, compared with those in the SCI group, *Nfκb2*, *Caspase-1*, and *Nlrp3* mRNA levels in the spinal cord were significantly greater in the SCI-DM group (Fig. 3G). These results indicate that pyroptosis is involved in the ability of T2D to hinder SCI recovery. Hereafter, we focused on the role of T2D on the microglial pyroptosis during SCI recovery.

### T2D induced excessive pyroptosis of microglia in the spinal cord of SCI mice

**Fig. 4.**
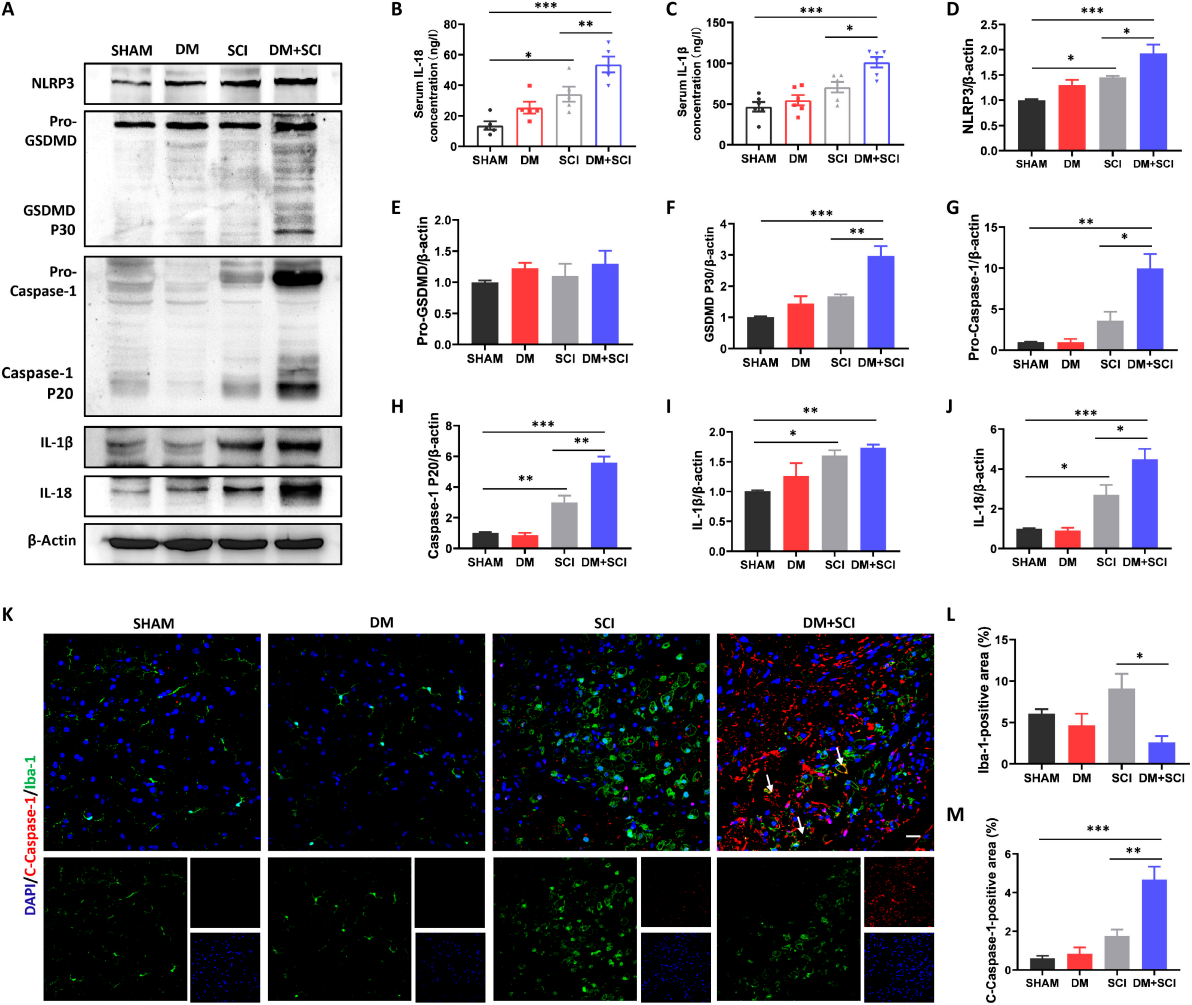
T2D-induced excessive pyroptosis of microglia in SCI mice. (A and D to J) Western blotting (WB) results and statistical analysis of the levels of NLRP3, IL-18, Caspase-1, IL-1β, and GSDMD in the spinal cord of mice on day 5 post-SCI (*n* = 3). (B and C) Levels of IL-18 and IL-1β in the serum (*n* = 5). (K to M) Costaining of C-Caspase-1 (red) and Iba-1 (green), and statistical analysis of C-Caspase-1- and Iba-1-positive areas in the spinal cord on day 5 post-SCI. Scale bar, 20 μm. *n* = 3. *P < 0.05, **P < 0.01, ***P < 0.001.

Pyroptosis is a type of cell death induced by inflammatory factors, which is involved in the SCI repair [25,26]. Here, we further explored whether excessive pyroptosis is important for triggering the loss of microglia in T2D combined with SCI. Compared with SCI, T2D significantly increased the expressions of GSDMD P30, Caspase-1 P20, IL-1β, IL-18, and NLRP3 in the spinal cord of SCI mice (Fig. 4A and D to J). Similarly, the levels of IL-18 and IL-1β in the serum were also significantly increased in the DM + SCI group (Fig. 4B and C). Furthermore, costaining for Cleaved (C)-Caspase-1 (labeled for pyroptosis) and Iba-1 (labeled for microglia) in the spinal cord revealed much stronger colocalization of C-Caspase-1 and Iba-1 in the spinal cord of the DM + SCI group (Fig. 4K to M). These results suggest that T2D induces increased microglial pyroptosis in the spinal cord after SCI.

**Fig. 5.**
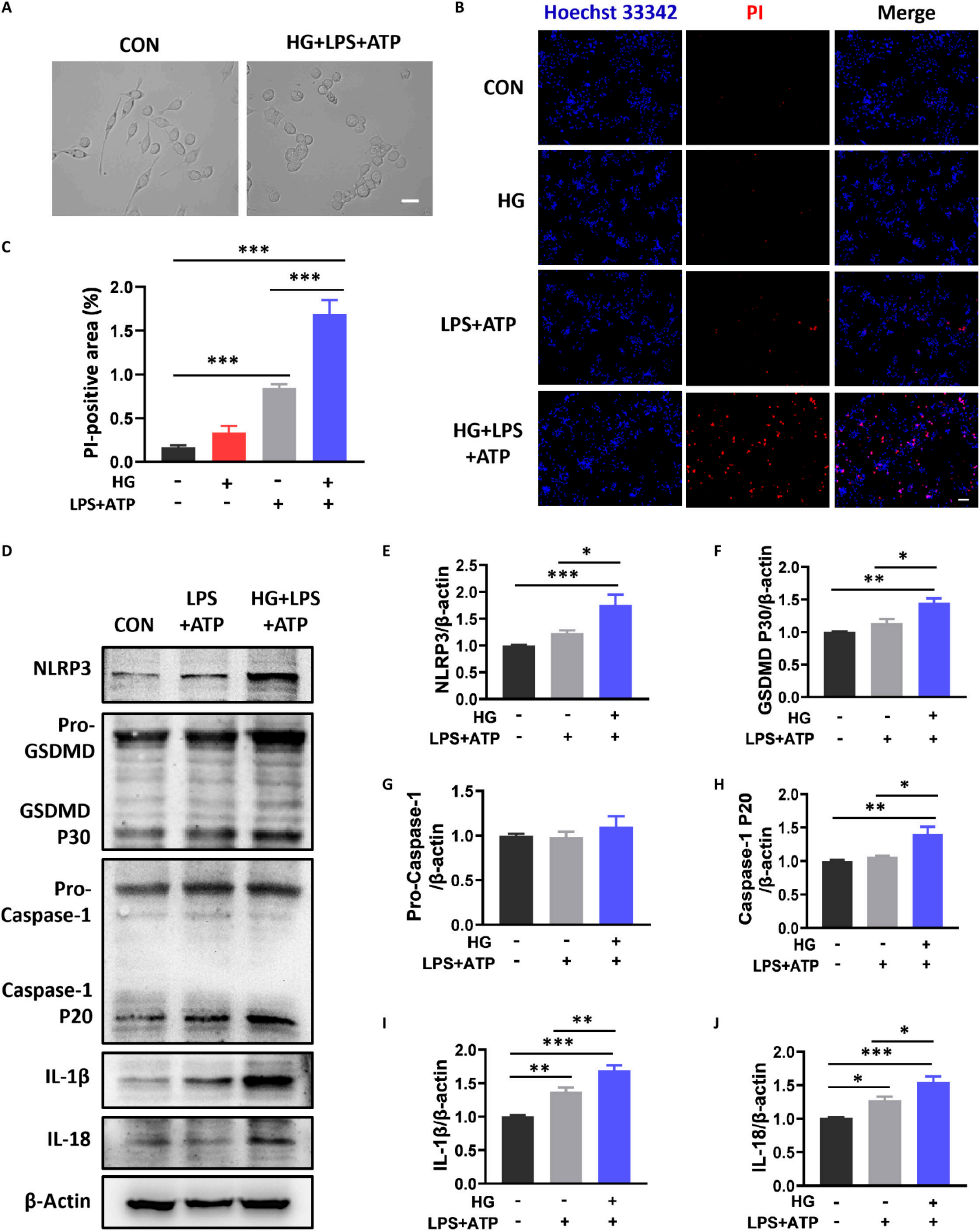
High glucose aggravated pyroptosis in BV-2 cells under LPS + ATP condition. (A) Morphology of BV-2 cells under different conditions. Scale bar, 50 μm. (B and C) Images of PI staining and statistical analysis of the PI-positive area. Scale bar, 50 μm. *n* = 5. (D to J) WB results and statistical analysis of NLRP3, GSDMD, Caspase-1, IL-18, and IL-1β (*n* = 3). *P < 0.05, **P < 0.01, ***P < 0.001.

To further prove the causal relationship between hyperglycemia and microglial pyroptosis, we used high glucose (HG) combined with lipopolysaccharide (LPS) + adenosine triphosphate (ATP) to stimulate the BV-2 cell lines to mimic the state of microglia in the context of T2D combined with SCI. As shown in Fig. 5A, BV-2 cells in the CON group presented a long strip morphology, whereas most BV-2 cells in the HG + LPS + ATP group presented a round and flattened morphology with unclear cytoplasm and swollen rupture. Hoechst 33342/propidium iodide (PI) costaining results also demonstrated that there were much more PI-positive BV-2 cells in the HG + LPS + ATP group (Fig. 5B and C). Additionally, consistent with the in vivo results, HG markedly increased expressions of the pyroptosis-related proteins in BV-2 cells after stimulation with LPS + ATP (Fig. 5D to J). Overall, HG aggravated pyroptosis of BV-2 cells under LPS + ATP condition.

### Conditional microglial *Caspase-1* gene knockout ameliorated nerve repair in T2D combined with SCI mice

**Fig. 6.**
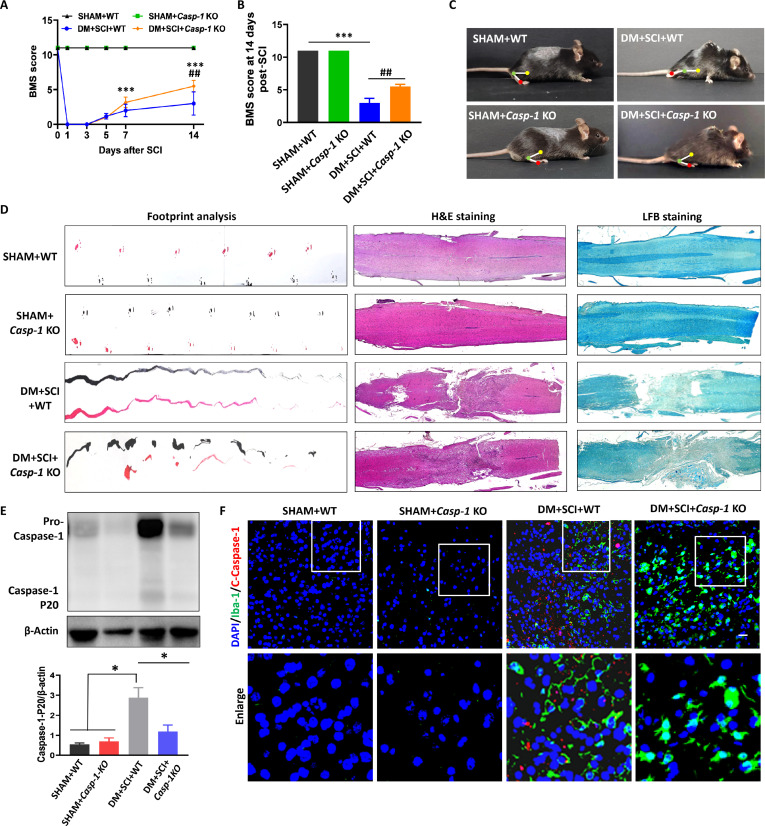
Conditional microglial *Caspase-1* gene KO ameliorated nerve repair in T2D combined with SCI mice. (A) BMS scores of the hind limbs of the mice on days 1, 3, 7, and 14 post-SCI (*n* = 6). (B) Statistical analysis of the BMS score on day 14 after SCI (*n* = 6). ***P < 0.001, DM + SCI + WT versus SHAM + WT; ^##^ *P* < 0.01, DM + SCI + WT versus DM + SCI + *Casp-1* KO. (C) Motion images of the mice on day 14 post-SCI (*n* = 6). (D) Footprints of the hind limbs (*n* = 6), H&E staining (*n* = 3), and LFB staining (*n* = 3) on day 14 post-SCI. (E) WB result and statistical analysis of the level of Caspase-1 in the spinal cord of mice on day 5 post-SCI (*n* = 3). *P < 0.05. (F) Costaining of C-Caspase-1 (red) and Iba-1 (green) in the spinal cord on day 5 post-SCI. Scale bar, 20 μm. *n* = 3.

To further reveal the role of microglial pyroptosis on nerve repair in T2D combined with SCI mice, we constructed a conditional microglial *Caspase-1* (*Casp-1*) gene knockout (KO) mouse model and a T2D combined with SCI mouse model. We found that there were no significant differences in the BMS scores, footprints, or histomorphology between the SHAM + wild type (WT) group and the SHAM + *Casp-1* KO group (Fig. 6A to D). However, compared with those in the SHAM + WT group, BMS scores of the mice in the DM + SCI + WT group and the DM + SCI + *Casp-1* KO group were significantly lower after SCI surgery and then increased gradually at 3 d post-SCI (Fig. 6A and B). Moreover, the BMS score in the DM + SCI + *Casp-1* KO group was much greater than that in the DM + SCI + WT group, especially at 14 d post-SCI (Fig. 6A and B). In addition, the hind limbs of the mice in the DM + SCI + *Casp-1* KO group exhibited much less persistent dragging, a much smaller damaged area, and greater remyelination of the spinal cord when compared with those in the DM + SCI + WT group (Fig. 6C and D). More importantly, *Casp-1* KO significantly decreased the expression of Caspase-1 expression in the spinal cord from DM + SCI + WT mice; the mice in the DM + SCI + *Casp-1*-KO group also presented weak colocalization of C-Caspase-1 and Iba-1 in the spinal cord than in the DM + SCI group (Fig. 6E and F). These results confirm that excessive pyroptosis of microglia is an important inducing event for more severe nerve damage in T2D combined with SCI mice.

### T2D promoted TXNIP–NLRP3 complex formation in SCI mice

**Fig. 7.**
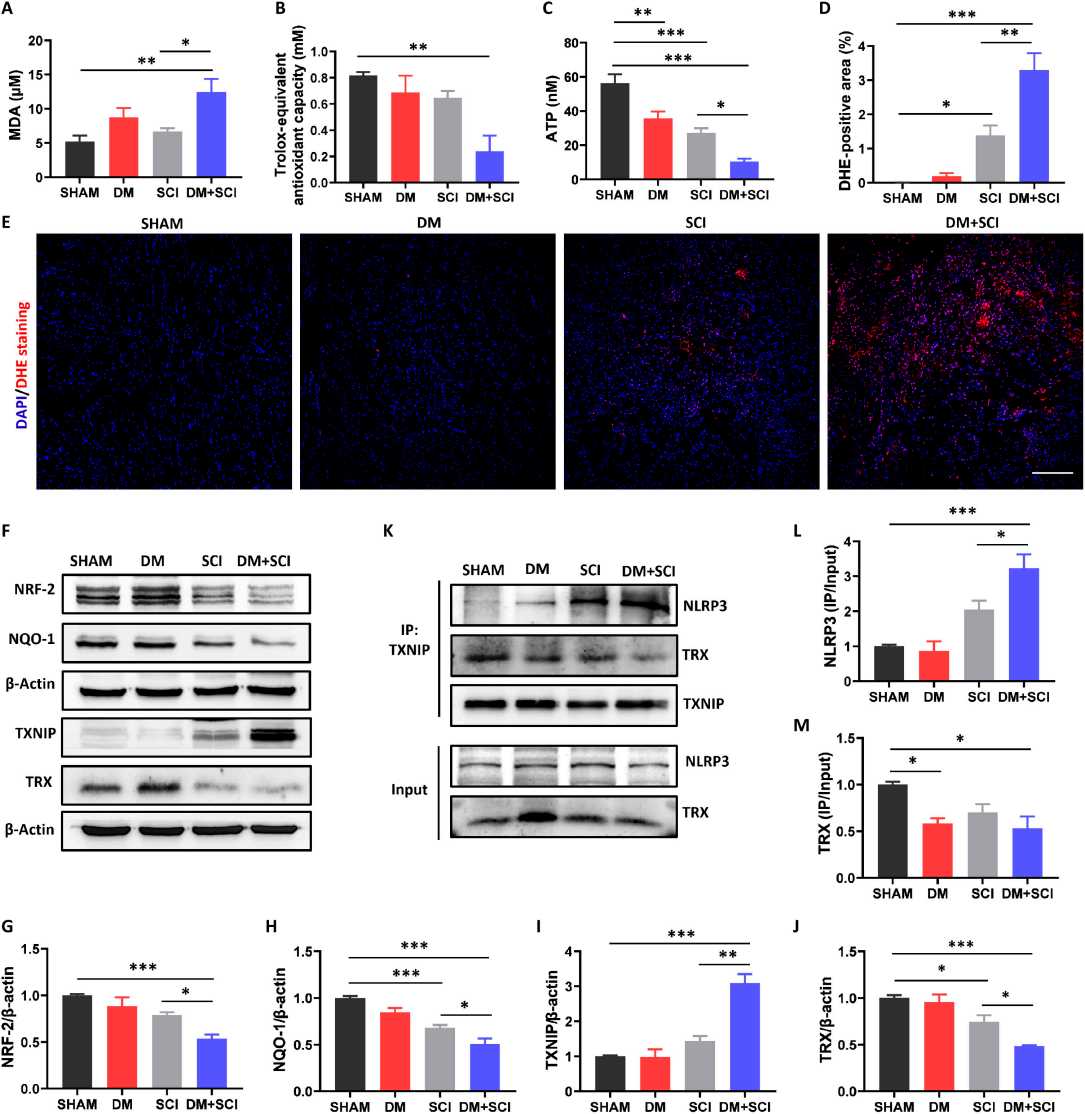
T2D promoted TXNIP–NLRP3 complex formation in SCI mice. (A) MDA content in the serum (*n* = 5). (B) Total antioxidant capacity in the serum (*n* = 3). (C) ATP content of the spinal cord (*n* = 5). (D and E) DHE staining and statistical analysis of the DHE-positive area in the spinal cord. Scale bar, 200 μm. *n* = 5. (F to J) WB results and statistical analysis of NRF-2, NQO-1, TXNIP, and TRX in the spinal cord of mice on day 5 post-SCI (*n* = 3). (K to M) Coimmunoprecipitation results and statistical analysis of the relationships among TXNIP, NLRP3, and TRX in the spinal cord of mice on day 5 post-SCI (*n* = 3). *P < 0.05, **P < 0.01, ***P < 0.001.

**Fig. 8.**
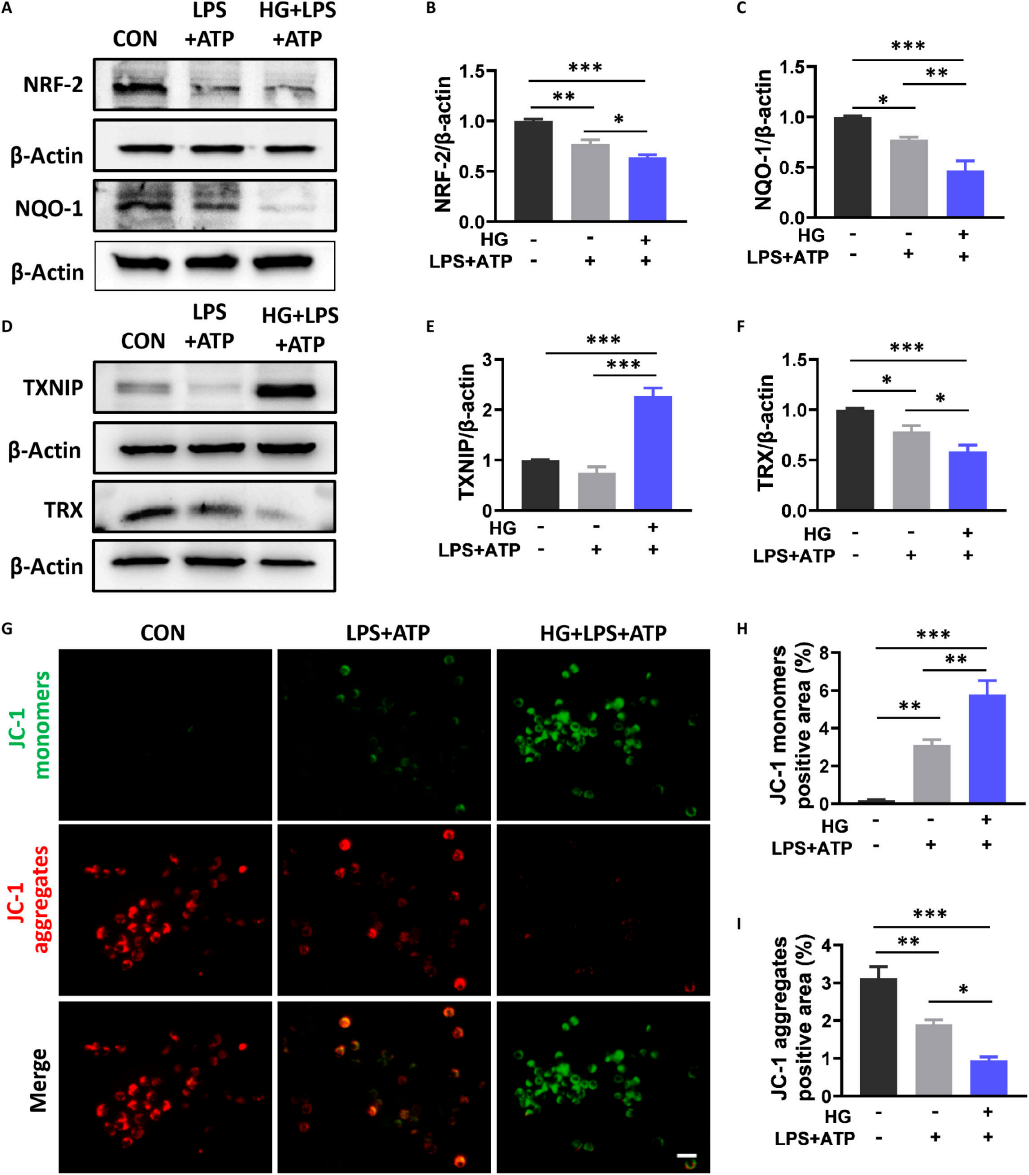
High glucose promoted mitochondrial dysfunction in BV-2 cells under LPS + ATP conditions. (A to F) WB results and statistical analysis of NRF-2, NQO-1, TXNIP, and TRX in BV-2 cells (*n* = 3). (G to I) Representative images of JC-1 staining and statistical analysis of JC-1 monomers and JC-1 aggregates. Scale bar, 50 μm. *n* = 5. *P < 0.05, **P < 0.01, ***P < 0.001.

Inflammasome activation is essential for initiating pyroptosis [20]. Mitochondrial dysfunction is a caused event for the activation of NLRP3 inflammasome [27]. Mitochondrial dysfunction results in a large amount of ROS production and then promotes the dissociation of TXNIP from TRX, and then TXNIP subsequently binds and activates the NLRP3 inflammasome [28]. Here, T2D was observed to significantly increase the malondialdehyde (MDA) level in the serum and suppress the total antioxidant capacity level in the serum and ATP level in the spinal cord of SCI mice (Fig. 7A to C). Dihydroethidium (DHE) staining also revealed many more DHE-positive signals in the spinal cord of the DM + SCI group (Fig. 7D and E). In addition, T2D remarkably suppressed the expression of antioxidant-related proteins (NRF-2, NQO-1, and TRX) and increased TXNIP expression in the spinal cord of SCI mice (Fig. 7F to J). Consistent with the in vivo results, the BV-2 cells in the HG + LPS + ATP group also presented increased expression of TXNIP and decreased expressions of NQO-1, NRF-2, and TRX (Fig. 8A to F). Moreover, the BV-2 cells in the HG + LPS + ATP group presented an inhibited mitochondrial membrane potential with the transformation of red fluorescent aggregates into green fluorescent monomers during JC-1 staining (Fig. 8G to I). Moreover, T2D significantly reduced the combination between TXNIP and TRX, and then TXNIP combined with NLRP3 in the spinal cord of SCI mice (Fig. 7K to M). These data indicate that T2D most likely promotes mitochondrial dysfunction and facilitates TXNIP–NLRP3 complex formation to activate the NLRP3 inflammasome in SCI mice.

### TXNIP inhibitor ameliorated T2D-induced the activation of NLRP3 inflammasome in SCI mice

**Fig. 9.**
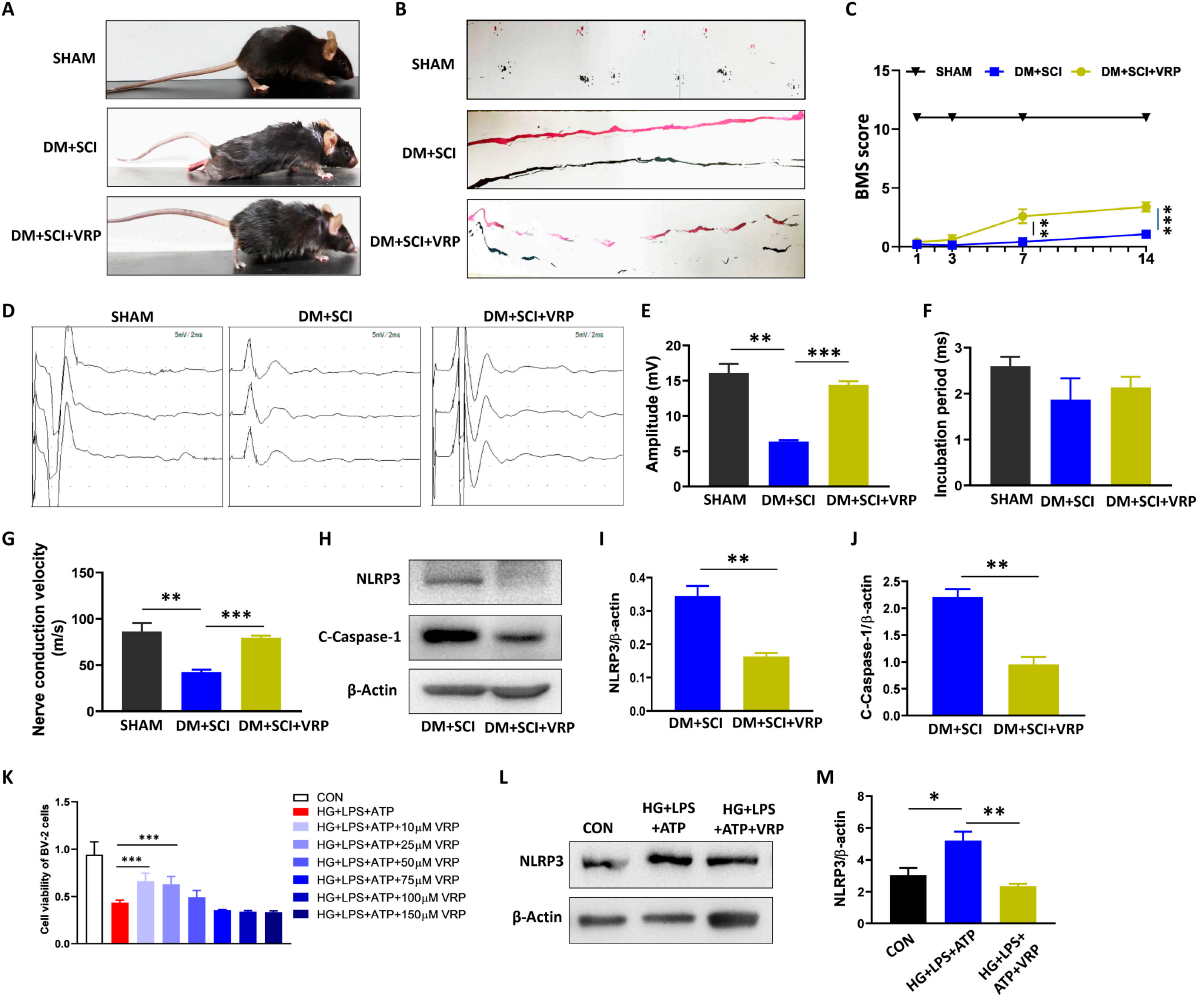
TXNIP inhibitor ameliorated T2D-induced activation of NLRP3 inflammasome in SCI mice. (A) Motion dynamic images of the mice (*n* = 6). (B) Footprints of the mice on day 14 post-SCI (*n* = 6). (C) BMS scores of the hind limbs of the mice on days 1, 3, 7, and 14 post-SCI (*n* = 6). Electrophysiological results (D) and analysis of amplitude (E), incubation period (F), and nerve conduction velocity (G) of electrical signals in hind limb of mice on day 14 post-SCI (*n* = 3). (H to J) WB results and statistical analysis of the levels of NLRP3 and C-Caspase-1 in the spinal cord of mice on day 5 post-SCI (*n* = 3). (K) Cell vitality of BV-2 cells during cell counting kit-8 (CCK-8) assay (*n* = 7). (L and M) WB result and statistical analysis of NLRP3 in BV-2 cells (*n* = 3). *P < 0.05, **P < 0.01, ***P < 0.001.

Here, we used the TXNIP inhibitor [Verapamil (VRP)] to further confirm that TXNIP is necessary for the activation of NLRP3 inflammasome during T2D hindering SCI recovery. The mice in the DM + SCI + VRP group presented a better locomotor functional recovery by easily lifting the instep of hind limbs, great BMS scores, and conspicuous breakpoints in the footprints (Fig. 9A to C). The electrophysiology results also demonstrated a significantly increased amplitude and fast nerve conduction velocity of hind limbs in the mice from the DM + SCI + VRP group (Fig. 9D to G). More importantly, VRP treatment significantly suppressed NLRP3 and C-Caspase-1 expressions in the spinal cords from DM + SCI mice (Fig. 9H to J). Consistent with the in vivo results, 10 μM VRP treatment also inhibited the activation of NLRP3 inflammasome and presented a stronger cell vitality in the BV-2 cells under HG + LPS + ATP condition (Fig. 9K to M). These results suggest that TXNIP is necessary for NLRP3 inflammasome activation during T2D hindering SCI recovery.

### The antioxidant NAC alleviated T2D-induced excessive pyroptosis of microglia in SCI mice

**Fig. 10.**
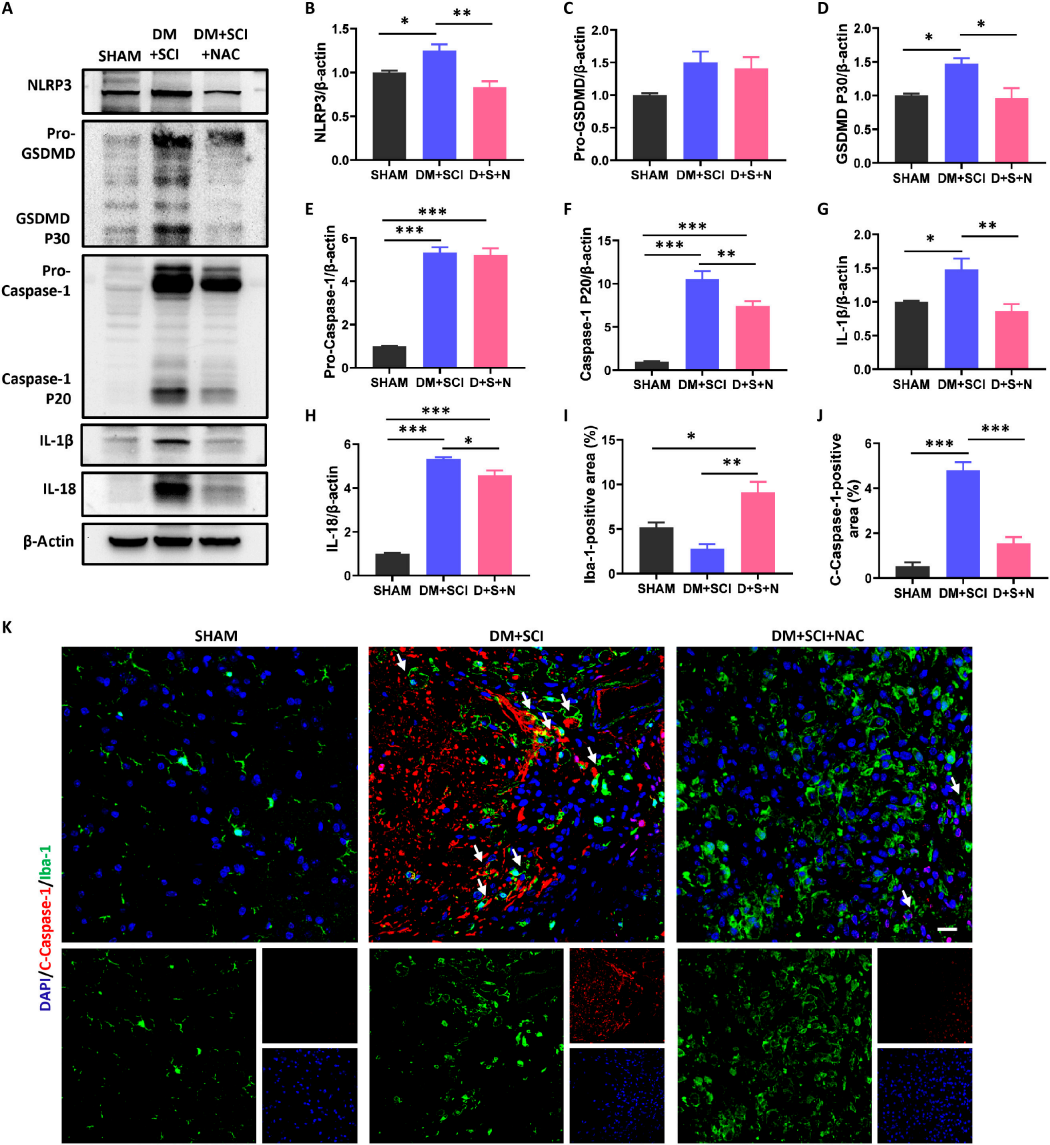
NAC treatment alleviated T2D-induced excessive pyroptosis of microglia in SCI mice. (A to H) WB results and statistical analysis of NLRP3, GSDMD, Caspase-1, IL-1β, and IL-18 in the spinal cord of mice on day 5 post-SCI (*n* = 3). (I to K) Costaining of C-Caspase-1 (red) and Iba-1 (green) and statistical analysis of positive signals of C-Caspase-1 and Iba-1 in the spinal cord of mice on day 5 post-SCI. Scale bar, 20 μm. *n* = 3. D + S + N, DM + SCI + NAC. *P < 0.05, **P < 0.01, ***P < 0.001.

Next, we used an antioxidant [*N*-acetyl-l-cysteine (NAC)] to further prove that elevated ROS are responsible for T2D-induced excessive microglial pyroptosis after SCI. The results revealed that NAC treatment significantly suppressed the expressions of NLRP3, GSDMD P30, Caspase-1 P20, IL-18, and IL-1β in the spinal cord of the DM + SCI group (Fig. 10A to H). Moreover, NAC treatment also remarkably decreased the colocalization of C-Caspase-1 and Iba-1 in the spinal cord of the DM + SCI group (Fig. 10I to K). In vitro, NAC treatment also inhibited the HG-triggered up-regulation in the expression of pyroptosis-related proteins and PI-positive signals in BV-2 cells under LPS + ATP condition (Fig. S2). These results verify that ROS exerts a vital role in T2D-induced excessive microglial pyroptosis in SCI mice.

### FPS-ZM1 treatment reduced the T2D-induced increase in ROS and microglial pyroptosis in SCI mice

**Fig. 11.**
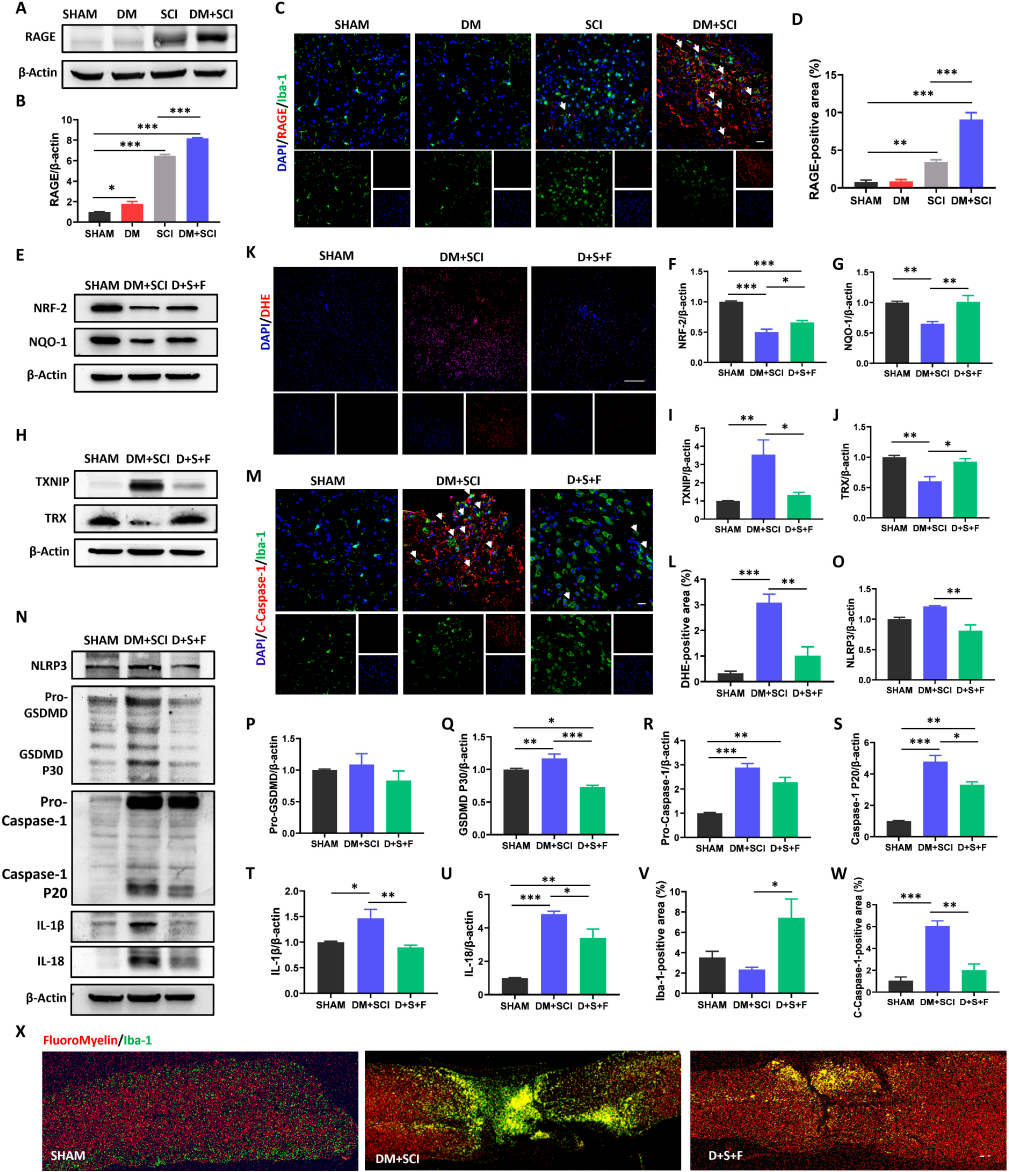
FPS-ZM1 treatment suppressed the T2D-induced increase in ROS and microglial pyroptosis in SCI mice. (A and B) WB result and statistical analysis of RAGE in the spinal cord of mice on day 5 post-SCI (*n* = 3). (C and D) Costaining of RAGE (red) and Iba-1 (green), and statistical analysis of positive signals of RAGE in the spinal cord on day 5 post-SCI. Scale bar, 20 μm. *n* = 3. (E to J) WB results and statistical analysis of NRF-2, NQO-1, TXNIP, and TRX in the spinal cord on day 5 post-SCI (*n* = 3). (K and L) DHE staining and statistical analysis of DHE-positive signals. Scale bar, 200 μm. *n* = 3. (M, V, and W) Costaining of C-Caspase-1 (red) and Iba-1 (green), and statistical analysis of positive signals of C-Caspase-1 and Iba-1 in the spinal cord of mice on day 5 post-SCI. Scale bar, 20 μm. *n* = 3. (N to U) WB results and statistical analysis of NLRP3, GSDMD, Caspase-1, IL-1β, and IL-18 in the spinal cord on day 5 post-SCI (*n* = 3). (X) Costaining of FluoroMyelin (red) and Iba-1 (green) in the spinal cord on day 14 post-SCI. Scale bar, 100 μm. *n* = 3. D + S + F, DM + SCI + FPS-ZM1. *P < 0.05, **P < 0.01, ***P < 0.001.

AGE-RAGE signaling is a common regulatory pathway for diabetes-related series of complications. KEGG pathway enrichment analysis indicated AGE-RAGE signaling involved in diabetes hindering SCI recovery (Fig. 3E). We speculated that AGE-RAGE signaling may be essential for the T2D-induced elevated ROS in SCI mice. We observed that the expression of RAGE in the spinal cords of the SCI and DM + SCI groups was greatly increased when compared with that of the SHAM and DM groups, and this phenomenon was much more pronounced in the DM + SCI group (Fig. 11A and B). Consistent with the WB result, costaining for Iba-1 and RAGE further revealed markedly increased expression of RAGE in microglia of the spinal cord from DM + SCI mice (Fig. 11C and D). Furthermore, we used a RAGE inhibitor [*N*-benzyl-4-chloro-*N*-cyclohexylbenzamide (FPS-ZM1)] to treat T2D combined with SCI mice and verified the role of RAGE. As expected, FPS-ZM1 treatment efficiently ameliorated T2D-induced excessive ROS and microglial pyroptosis both in vivo and in vitro (Fig. 11E to W and Fig. S3). We also observed the decreases of demyelinating lesion volume and Iba-1^+^ cell density in FPS-ZM1-treated DM + SCI mice on day 14 post-SCI, indicating the better remyelination in DM + SCI + FPS-ZM1 mice compared with DM + SCI mice (Fig. 11X). These data suggest that RAGE signaling triggers the excessive pyroptosis of microglia induced by T2D via increased ROS level.

### Both NAC and FPS-ZM1 promoted nerve repair in T2D combined with SCI mice

**Fig. 12.**
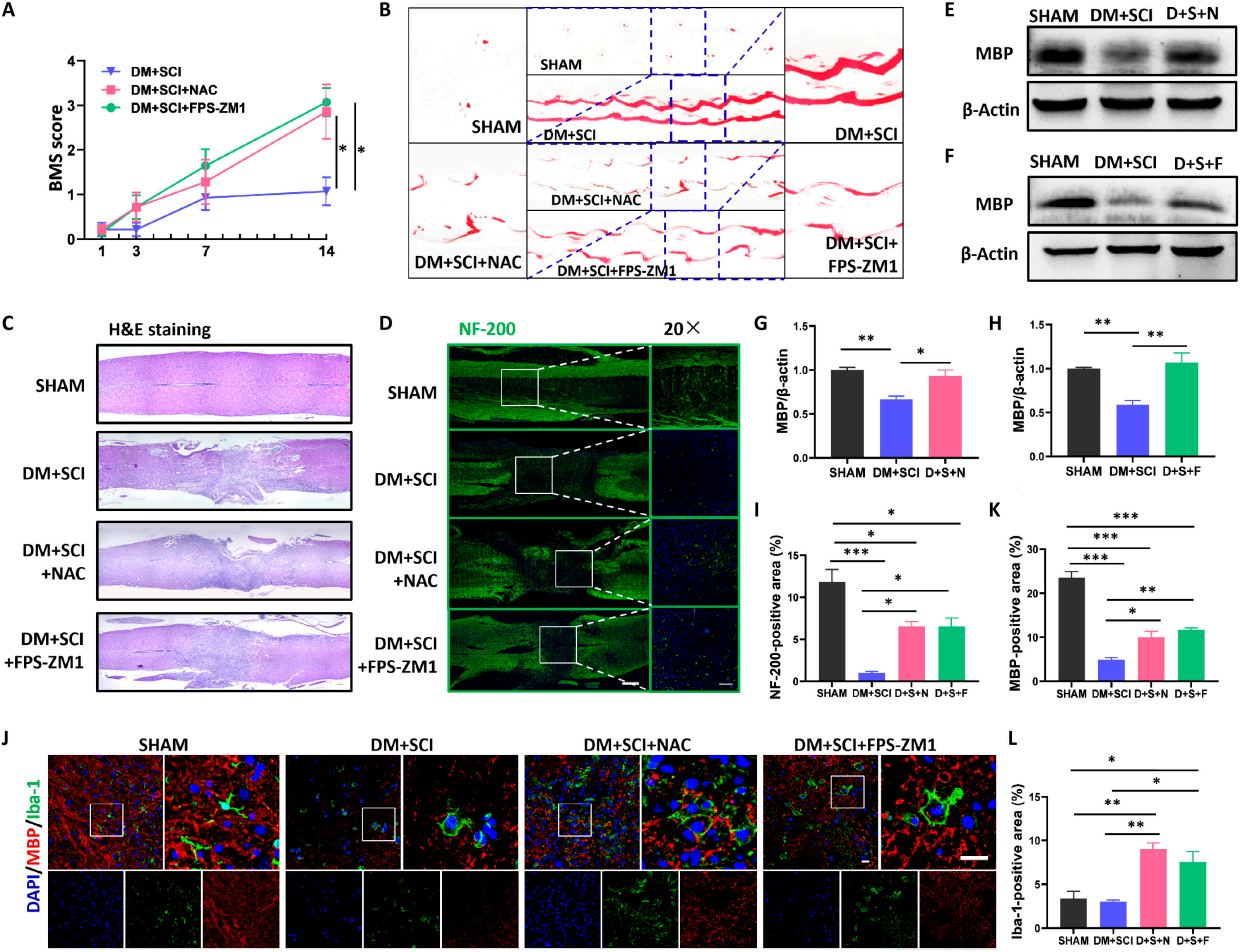
Both NAC and FPS-ZM1 treatment promoted nerve repair in T2D combined with SCI mice. (A) BMS scores of the hind limbs of mice (*n* = 7). (B) Footprints of the hind limbs (*n* = 6). (C) H&E staining results of longitudinal sections of the spinal cord (*n* = 3). (D and I) Immunofluorescence staining of NF-200 (green) and statistical analysis of NF-200-positive signals in the injured spinal cord of mice on day 14 post-SCI. Scale bar, 100 μm. *n* = 3. (E to H) WB results and statistical analysis of MBP in the spinal cord on day 5 post-SCI (*n* = 3). (J to L) Costaining of MBP (red) and Iba-1 (green), and statistical analysis of positive signals of MBP and Iba-1 in the spinal cord on day 5 post-SCI. Scale bar, 20 μm. *n* = 3. D + S + N, DM + SCI + NAC; D + S + F, DM + SCI + FPS-ZM1. *P < 0.05, **P < 0.01, ***P < 0.001.

Finally, we explored the effects of NAC and FPS-ZM1 treatment on nerve repair in T2D combined with SCI mice. NAC and FPS-ZM1 treatment both increased the BMS scores of the mice in the DM + SCI group in a linear relationship, especially on day 7 post-SCI (Fig. 12A). Moreover, there were conspicuous breakpoints rather than continuous drag in the footprints of hind limbs in the mice from the NAC- or FPS-ZM1-treated DM + SCI groups, indicating that the locomotor function of the hind limbs in the mice from these 2 groups had partly recovered (Fig. 12B). Furthermore, both NAC and FPS-ZM1 treatment promoted many more neurofilaments to cross the damaged area of the spinal cord and decreased the injured area of the spinal cord in DM + SCI mice (Fig. 12C, D, and I). In addition, NAC and FPS-ZM1 treatment not only significantly increased the number of microglial vesicles and improved their phagocytic function, but also increased MBP expression in the spinal cord of the DM + SCI group (Fig. 12E to H and J to L). These data demonstrate that the activation of the RAGE–ROS–NLRP3 axis is crucial to the microglial pyroptosis and poor remyelination in T2D combined with SCI mice.

## Discussion

Nerve damage is an important and common complication caused by diabetes [29]. Diabetes has been reported to seriously impede nerve recovery after SCI [3,7,18]. In this study, a T2D combined with SCI mouse model was established to confirm that T2D is detrimental to SCI recovery and can even lead to more severe mortality in SCI mice. Here, we focused on the poor microglial phagocytic function and explored the related regulatory mechanism for the difficulty in nerve repair of diabetes combined with SCI. Mechanistic studies revealed that T2D increases ROS by activating the AGE-RAGE pathway, thereby promoting the formation of the TXNIP–NLRP3 complex to activate NLRP3 inflammasome and induce microglial pyroptosis. These effects hinder the phagocytizing function of microglia to clear myelin debris and impede remyelination and nerve regeneration after SCI.

There are many studies confirming that diabetes hinders SCI recovery; however, most studies focus on the functional recovery of neurons or endothelial cells (ECs) after SCI [3,18,30]. For microglia, they mainly focus on the inflammatory response caused by microglial activation and its influence on neuronal function under diabetic condition. Except for regulating inflammatory response, the most important role of microglia is that they act as phagocytes to clear myelin debris and provide a favorable microenvironment for remyelination after SCI. The presence of myelin debris hinders the remyelination process during SCI recovery [2,31]. Accordingly, timely removal of myelin debris is important for SCI recovery. The present work demonstrated that T2D induces the loss of microglia and leads to a poor phagocytizing function of microglia for clearing myelin debris after SCI. These results suggest that loss of microglia is a cause of T2D-induced poor remyelination and nerve repair in SCI mice.

Diabetes is associated with a series of complications [32–34]. Excessive cell death is a common mechanism for diabetes-associated complications. Diabetes triggers the apoptosis of neurons and ECs in the spinal cord from SCI mice [3,35]. Our RNA-Seq results suggest that inflammatory response and pyroptosis are also essential for the difficulty of diabetes combined with SCI repair. As a type of innate immune cells in central nervous system, microglia not only release the inflammatory factors to exacerbate secondary injury of SCI, but also are affected by inflammatory factors [36,37]. Pyroptosis is also known as inflammatory necrosis of cells [38,39]. As such, we speculated that in addition to apoptosis, pyroptosis may be a new mechanism by which T2D triggers the loss of microglia. This notion was supported by our results showing that T2D significantly activates the NLRP3 inflammasome and triggers pyroptosis, with increased expression of pyroptosis-related proteins. Importantly, these findings and conclusions are further validated in microglia-specific *Caspase-1* KO mice. *Caspase-1* KO promoted nerve repair in T2D combined with SCI mice.

Studies in humans and type 1 diabetes (T1D) mouse model demonstrated that hyperglycemia increases inflammatory responses in microglial cells via NF-κB activation and exacerbates secondary injury, thus resulting in a poor outcome after SCI [18]. Our previous works have also demonstrated that T1D greatly exacerbates the loss of ECs and destroys the integrity of BSCB after SCI [3,11]. Compared with T1D, T2D has more complex pathological features. T1D and T2D may exert different effects on the function of microglia during a specific pathological process of SCI. Moreover, different pathological stages of T2D may also have different effects on the neural repair of SCI. In this study, we mainly revealed the effect of T2D on the phagocytic function of microglia in the early stage of SCI. Neuroinflammation runs through the entire repair process of SCI. Thus, we may need to pay more attention to the influence of T2D on neuroinflammation caused by microglia in the later stage of SCI.

The NLRP3 inflammasome exists in microglia that can promote the activation of Caspase-1 and induce the pyroptosis of microglia/macrophages after SCI [40]. Inhibiting its activation in microglia promotes neural repair after SCI [41]. We observed that T2D significantly induced mitochondrial dysfunction and promoted TXNIP to bind and activate NLRP3 inflammasome after SCI. Notably, VRP treatment further demonstrated that TXNIP is necessary for activating NLRP3 inflammasome. Suppressing ROS production by NAC significantly attenuated T2D-associated activation of the NLRP3 inflammasome and excessive pyroptosis in microglia, which promoted myelin clearance and nerve regeneration after SCI. It is worth noting that in addition to the NLRP3 inflammasome, IL-1β level was significantly increased in the serum samples, as well as in the spinal cords from the DM + SCI group in RNA-Seq and WB results. This result suggests that NLRP3 or IL-1β may serve as important indicators for the clinical prognosis of T2D combined with SCI patients. Taken together, excessive pyroptosis is a critical causal event by which T2D induced loss of microglia during SCI recovery.

Chronic hyperglycemia causes a variety of proteins to undergo the Maillard reaction, followed by the formation of many AGEs [42]. With the continuous accumulation of endogenous or exogenous AGEs, a series of diabetic complications, including diabetic neuropathy, are triggered when AGEs act on RAGE [43]. AGEs that bind with RAGE can activate the intracellular signaling pathways, generate oxidative stress, and release proinflammatory cytokines [44]. Blocking RAGE is promising for preventing diabetes complications [45]. This is as we have demonstrated herein. T2D significantly enhanced RAGE expression in microglia of the spinal cord after SCI. Suppressing RAGE with FPS-ZM1 markedly ameliorated T2D-induced excessive ROS production and subsequent pyroptosis of microglia. These results further explain why T2D induces the overproduction of ROS to trigger the activation of NLRP3 inflammasome. Of course, using the specific NLRP3 inhibitor, such as MCC950, to verify the effect of FPS-ZM1 or NAC on the activation of NLRP3 inflammasome in neural repair of T2D combined with SCI mice can promote their role in clinical transformation. This is an important part of our research in future. In conclusion, these molecular events block myelin clearance and nerve regeneration in T2D combined with SCI mice.

However, there are some shortcomings that restrict the clinical translation of this study. Microglia are not a homogeneous cell population but encompass multiple functionally distinct subtypes. Although Iba-1 is widely used as a marker for the activation of microglia, it is better to further analyze the heterogeneity of microglial subtypes in the T2D combined with SCI mice, which will further deepen the regulatory theory of diabetes on microglia. Additionally, although both NAC and FPS-ZM1 alleviated microglial pyroptosis and promoted the locomotor functional recovery in T2D combined with SCI mice, the methods used to administer NAC and FPS-ZM1 in vivo lack targeting properties. Although we have tried to explore the direct role of FPS-ZM1 and NAC on microglia in vitro, there is a need to further use the conditional microglial *Rage* gene or ROS-related gene KO mouse model to verify this conclusion. These are the scientific issues that we will explore in future.

**Fig. 13.**
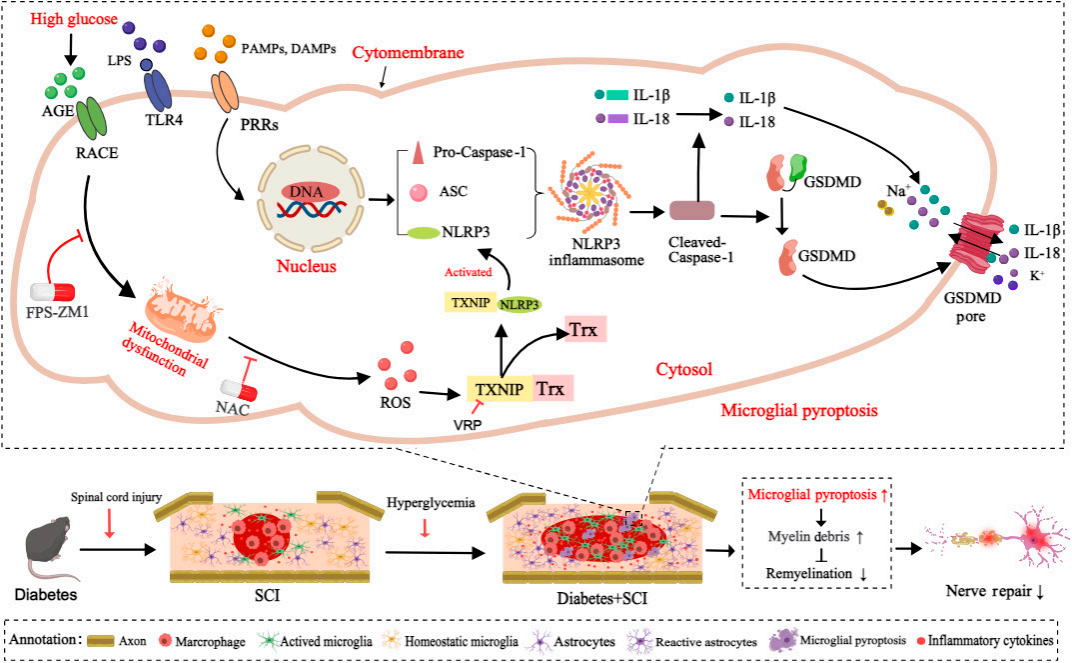
Schematic diagram depicting the role and mechanism by which T2D triggers excessive pyroptosis of microglia in SCI mice. After SCI, T2D greatly induces mitochondrial dysfunction by activating RAGE signaling to promote ROS production and trigger TXNIP dissociation from TRX. TXNIP then binds and activates the NLRP3 inflammasome in microglia. The activation of the NLRP3 inflammasome induces excessive microglial pyroptosis and suppresses its phagocytizing function, thereby leading to severe accumulation of myelin debris and poor remyelination of the spinal cord.

In conclusion, this study provides evidence that T2D significantly induces mitochondrial dysfunction by activating AGE-RAGE signaling to promote ROS production. The overproduction of ROS triggers TXNIP to dissociate from TRX and then bind to and activate the NLRP3 inflammasome in microglia. Importantly, this effect further induces the microglial pyroptosis and suppresses its phagocytizing function to clear myelin debris, thereby leading to poor remyelination in SCI mice (Fig. 13). Therefore, RAGE–ROS–NLRP3 axis-mediated microglial pyroptosis may be a novel therapeutic target for T2D combined with SCI recovery.

## Materials and Methods

### Animals and ethics statement

Eight-week-old male C57BL/6J mice (Ziyuan Laboratory Animal Technology, Hangzhou, China) were purchased and housed in a standard condition with a 12-h light/12-h dark cycle, humidity (60%), and an appropriate temperature (22 °C). The mice freely received water and food. All experimental procedures were approved by the Laboratory Animal Ethics Committee of Wenzhou University according to the National Institutes of Health Guide. The ethical approval number is WZU-2022-022.

### Generation of T2D combined with SCI mouse model

The mice were randomly divided into the normal diet (ND) group and the high-fat diet (HFD) group. In the HFD group, the mice were fed a 60% fat diet for 6 weeks and then intraperitoneally injected with STZ (75 mg/kg) for 2 d to induce the T2D diabetic model. We monitored random blood glucose levels in the mice at 3 d post-STZ injection to assess whether the blood glucose values of mice had increased. After 1 week, the mice whose blood glucose value was ≥16.7 mM for 3 consecutive days were identified as T2D mice. The mice in the ND group were fed with normal diet and intraperitoneally injected with the same amount of normal saline at the corresponding time points. Subsequently, the mice in the ND group were randomly divided into the SHAM group and SCI group; the mice in the T2D group were randomly divided into the DM group, DM + SCI group, DM + SCI + NAC group, DM + SCI + FPS-ZM1 group, and DM + SCI + VRP group. The sample sizes between the experiments were calculated using G*Power to ensure the reasonability. For SCI surgery, the mice were anesthetized, and a laminectomy was then performed at the T9 level to expose the spinal cord. The exposed spinal cord tissue was then hit with an Allne striker. The mice were considered as a successful SCI model because of the following phenomena: (a) the spinal cord immediately experienced edema and congestion; (b) the motor function of the hind limbs was impaired after awakening from anesthesia, with a BMS score = 0, and urinary retention occurred; (c) both hind limbs and the tail dragged on the ground. The mice in the DM + SCI + NAC group and DM + SCI + FPS-ZM1 group were intraperitoneally injected with NAC (100 mg/kg) and FPS-ZM1 (1 mg/kg), respectively, every 2 d from the successful establishment of T2D to the time of sacrifice. The mice in the DM + SCI + VRP group received an in situ spinal cord injection of VRP (100 mg/kg) once after the SCI surgery. The mice in the SHAM and DM groups all performed the same surgical procedure but without impact injury from Allne striker.

### Generation of conditional microglial Caspase-1 gene KO mice

In accordance with the mRNA sequence of *Caspase-1* gene (National Center for Biotechnology Information GenBank, gene number: 12362), a conditional microglial *Caspase-1* gene KO mouse was generated via CRISPR/Cas9 technology (Cyagen, Suzhou, China). Specifically, the guide RNA target sequence of the *Caspase-1* gene was designed, obtained, and then injected into fertilized eggs to obtain F0 mice. After that, F0 mice were identified via polymerase chain reaction (PCR) or sequencing and bred with WT mice to obtain F1 mice, and the PCR and sequencing were used to identify the genotype of the F1 mice. Finally, the heterozygous F1 mice were bred and genotyped to obtain *Caspase-1* Flox/Flox mice. Subsequently, *Caspase-1* Flox/Flox mice were induced into conditional microglial *Caspase-1* gene KO mice by injecting the iCRE-WT tool virus (a type of adenovirus-related virus from GENE, Shanghai, China).

### Locomotor function assessment

The locomotor function of mice was evaluated via the footprint analysis and BMS score. For BMS score, the mice moved freely in an open field for 5 min at 1, 3, 7, and 14 d post-SCI. Then, the operator carefully evaluated the ankle joint movement, instep standing and turning over, and coordinated the hind limbs of the mice. The locomotor function of the mice was analyzed according to a BMS score ranging from 0 (no limb movement or weight support) to 11 (normal locomotion). For footprint analysis, the hind limbs of mice were dipped in red dye or black dye and then went across a narrow channel (1 m long and 7 cm wide). Finally, the footprints were scanned into images and then applied to analyze the locomotor function of mice. For electrophysiology assessment, the spinal cord of mice was pried at the C2–C3 segment after nanesthesis, and the exposed spinal cord was connected with the current input end through the needle. Then, the gastrocnemius muscle of hind limb was exposed and connected with the current outgoing end through the needle. Then, the C2–C3 segment of the spinal cord was stimulated with the same intensity of electricity using electromyography/evoked potential instrument (NeuroExam M-800A, MEDCOM, China). The electricity passed through the injured or uninjured T9–T10 segment. After stimulating with the same intensity for 3 times, the amplitude, incubation period, and nerve conduction velocity were analyzed in the electric wave motion.

### LFB staining

The myelin structure of the spinal cord in the mice was evaluated by an LFB staining kit. The 5-μm longitudinal spinal cord sections were dewaxed and stained with LFB staining solution (Jingke Bio, WB1030). The LFB staining kit included myelin staining solutions A, B, and C. Firstly, the sections were incubated with preheated solution A and then alternately differentiated with solution B and solution C until the background of the myelin sheath of the spinal cord was nearly colorless. Lastly, the images were captured under an optical microscope.

### H&E staining and Nissl staining

The spinal cord of the mice was obtained and fixed with 4% paraformaldehyde at 14 d post-SCI. After dewaxing and rehydration, transverse or longitudinal spinal cord sections were incubated with the corresponding hematoxylin and eosin (H&E) staining reagents (Beyotime, C0105S) and Nissl staining kits (Beyotime, C0117) following the manufacturers’ instructions. Finally, the images were captured via an optical microscope (Leica, Germany).

### Immunofluorescence staining

After dewaxing and rehydration, transverse or longitudinal spinal cord sections were incubated with 3% H_2_O_2_ to block endogenous peroxidase activity for 15 min at room temperature. Next, the sections were incubated with 5% bovine serum albumin for 30 min and then incubated with the following primary antibodies for overnight at 4 °C: NF-200 (1:200, 18934-1-AP), Iba-1 (1:200, Ab178847), MBP (1:200, Sc-271524), RAGE (1:200, Ab216329), and C-Caspase-1 (1:200, 22915-1-AP). The sections were subsequently incubated with goat anti-mouse or anti-rabbit IgG H&L (Alexa Fluor 594 and Alexa Fluor 488) for 2 h. For costaining of FluoroMyelin and Iba-1, after incubation with Iba-1 and related secondary antibody, the sections were washed with PBS and incubated with FluoroMyelin (1:400, F34652) for 20 min. Then, the sections were stained with 4′,6-diamidino-2-phenylindole (DAPI) to label the nuclei and subsequently observed in a laser scanning confocal microscope (Olympus, Japan). The images were quantified via ImageJ software. The number of positive cells was visualized and analyzed in 5 randomly selected fields per section at ×400 magnification via Image-Pro Plus.

### DHE staining

Here, we used DHE staining to evaluate the superoxide level in the spinal cord tissue of mice. Briefly, the spinal cord tissues were embedded in optimal cutting temperature (OCT) and then cut into 5-μm transverse spinal cord sections. The sections were incubated with DHE reagent (Beyotime, S0063) according to the manufacturer’s instructions and then stained with DAPI to label the nuclei. Finally, the DHE-positive signals in the spinal cord were visualized under a laser scanning confocal microscope and analyzed via ImageJ software.

### Microglial cell line (BV-2 cells) culture and treatment

BV-2 cells were purchased from the cell storage center of Wuhan University (Wuhan, China) and then cultured in Dulbecco’s modified Eagle’s medium supplemented with 1% streptomycin/penicillin and 10% fetal bovine serum at 37 °C in 5% CO_2_. The BV-2 cells were divided into 6 groups: the control (CON), LPS + ATP, HG + LPS + ATP, HG + LPS + ATP + NAC, HG + LPS + ATP + FPS-ZM1, and HG + LPS + ATP + VRP groups. BV-2 cells were pretreated with FPS-ZM1 (500 nM), NAC (3 mM), or VRP (10, 25, 50, 75, 100, and 150 μM) in culture medium for 1 h and then treated with HG medium (75 mM) containing LPS (1 μg/ml). Finally, the BV-2 cells were treated with ATP (1 mM) for 1 h and then collected for biochemical and molecular biology analyses.

### WB analysis

BV-2 cells and spinal cord segments including the lesion center were collected and dissociated with radioimmunoprecipitation assay (RIPA) lysis buffer. The protein mixture was subsequently extracted and quantified via a bicinchoninic acid (BCA) assay. After that, equal amounts of protein were separated by 8% or 12% sodium dodecyl sulfate–polyacrylamide gel electrophoresis and then transferred to polyvinylidene difluoride membranes. The membranes were blocked with 5% nonfat milk for 1 h and then incubated with the following primary antibodies overnight at 4 °C: MBP (1:1,000, Sc-271524), Iba-1 (1:1,000, Ab178847), NRF-2 (1:1,000, 16396-1-AP), IL-18 (1:1,000, 10663-1-AP), NQO-1 (1:1,000, Ab80588), TXNIP (1:1,000, CST14715), TRX (1:1,000, 14999-1-AP), Caspase-1 (1:1,000, 22915-1-AP), C-Caspase-1 (1:1,000, Sc-398715), NLRP3 (1:1,000, Ab263899), GSDMD (1:1,000, CST10137), and IL-1β (1:1,000, 16806-1-AP). The next day, the membranes were washed with TBST (tris-buffered saline with 0.05% Tween 20) for 3 times and then incubated with the appropriate secondary antibodies for 4 h at room temperature. Finally, the signals were visualized under a ChemiDocXRS Imaging System (Bio-Rad), and the densitometric values of protein expression were statistically analyzed via ImageJ software. β-Actin (Affinity, T0022) was used as a loading control. All the experiments were performed 3 times with the different tissues from different mice or different cells from different culture holes.

### RNA-Seq analysis

The differential gene expression in the spinal cord tissue was analyzed by RNA-Seq analysis (Shanghai OE Biotech Co. Ltd.). Briefly, the tissues were collected and immediately stored at −20 °C. Total RNA was extracted and then reverse transcribed into cDNA. The cDNA samples were subsequently sequenced via the Illumina NovaSeq 6000 system. Finally, we performed Venn diagram analysis, volcano plot analysis, PCA, GO enrichment analysis, and KEGG enrichment analysis of the quantitative and standardized samples.

### Assessment of total antioxidant capacity and MDA levels in the serum

The total antioxidant capacity and lipid peroxidation products in the serum of each mouse were assessed via a total antioxidant capacity detection kit (Beyotime, S0121) and a lipid peroxidation product (MDA) assay kit (Beyotime, S0131S) according to the manufacturer’s instructions.

### Assessment of ATP levels

The ATP levels in spinal cord tissue were detected by an ATP assay kit (Beyotime, S0026). Briefly, the spinal cord was incubated with lysis buffer and homogenized with a homogenizer. After centrifugation at 12,000*g* for 5 min at 4 °C, the protein from the spinal cord was extracted and collected for subsequent determination. Then, 20 μl of equal amounts of protein was incubated with 100 μl of ATP detection working solution for 3 min at room temperature. Lastly, the ATP level was quantified with a microplate reader.

### Mitochondrial membrane potential assay (JC-1 staining)

The mitochondrial function of BV-2 cells was assessed via JC-1 staining (Beyotime, C2006). Briefly, the BV-2 cells were rinsed with phosphate-buffered saline (PBS) for 3 times and then stained with working solution for 20 min at 37 °C. Then, images were captured with a fluorescence microscope (Carl Zeiss, Germany). Using the Image-Pro Plus, the number of JC-1 monomer- or JC-1 aggregate-positive cells was visualized and analyzed in 5 randomly selected fields per section at ×400 magnification.

### Coimmunoprecipitation

Spinal cord segments including the lesion center were dissociated with RIPA lysis buffer. The protein mixture was subsequently extracted and quantified via a BCA assay. After that, equal amounts of protein were incubated with anti-TXNIP (1:50, Cell Signaling Technology, 14715) overnight at 4 °C. The next day, BeyoMag Protein A+G Magnetic Beads (Beyotime, P2108) were added to the protein mixture and incubated for 3 h at room temperature. Finally, the proteins were eluted from the magnetic beads and analyzed via WB.

### Statistical analyses

All experiments and statistical analyses were performed in a double-blinded manner. All the results were shown as the means ± standard error of the mean (SEM) and analyzed via GraphPad Prism 8 and ImageJ. *T* test was performed to analyze the statistical significance between 2 independent groups. When the data were normally distributed, one-way analysis of variance (ANOVA) followed by Tukey’s multiple comparison test was performed to analyze the differences among 3 or more groups. When the data were not normally distributed, the nonparametric Mann–Whitney *U* test was used to analyze the differences. *P* < 0.05 indicated a statistically significant difference. Using different spinal cord tissues or cells, all the experiments were performed at least 3 times with consistent conditions to ensure accuracy.

## Ethical Approval

Animal experiments in this study were approved by the Laboratory Animal Ethics Committee of Wenzhou University according to the National Institutes of Health Guide (approval number: WZU-2022-022).

## Data Availability

The data that support the findings of this study are available from the corresponding authors upon reasonable request.
