# Supplementary material for: Type 2 Diabetes Promotes the Microglial Pyroptosis by Activating NLRP3 Inflammasome to Impede Remyelination After Spinal Cord Injury
Source: Research (Wash D C). 2026 Apr 14;9:1237. doi: 10.34133/research.1237 (PMC13077133; doi:10.34133/research.1237)
Supplement: Supplementary 1 — Figs. S1 to S3 [file research.1237.f1.zip › Supplementary figures.docx]

**Supplementary Figures**

**Figure S1**

**
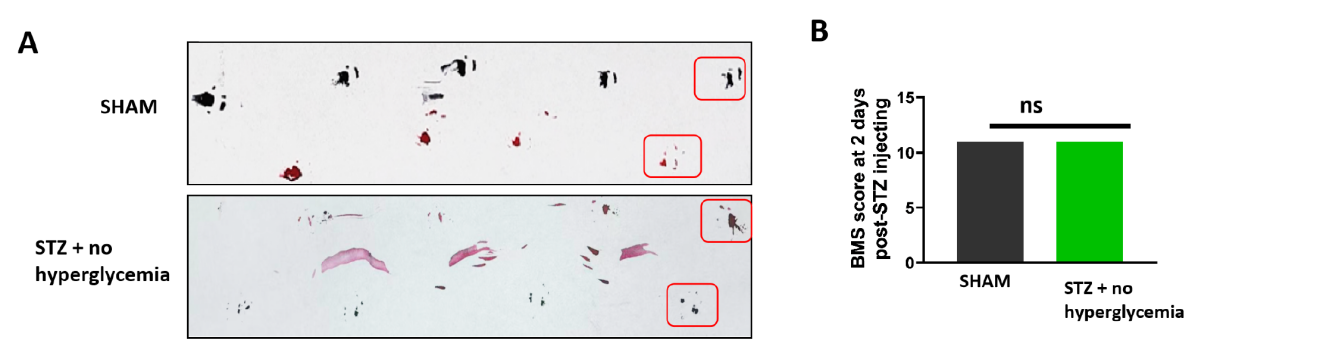
**

**Figure S1** The potential effect of STZ toxicity on locomotor function of mice. (A) Footprints of the mice on day 7 post-STZ injecting, n=6. (B) BMS score of the hindlimbs of the mice on day 7 post-STZ injecting, n=6.

**Figure S2**

**
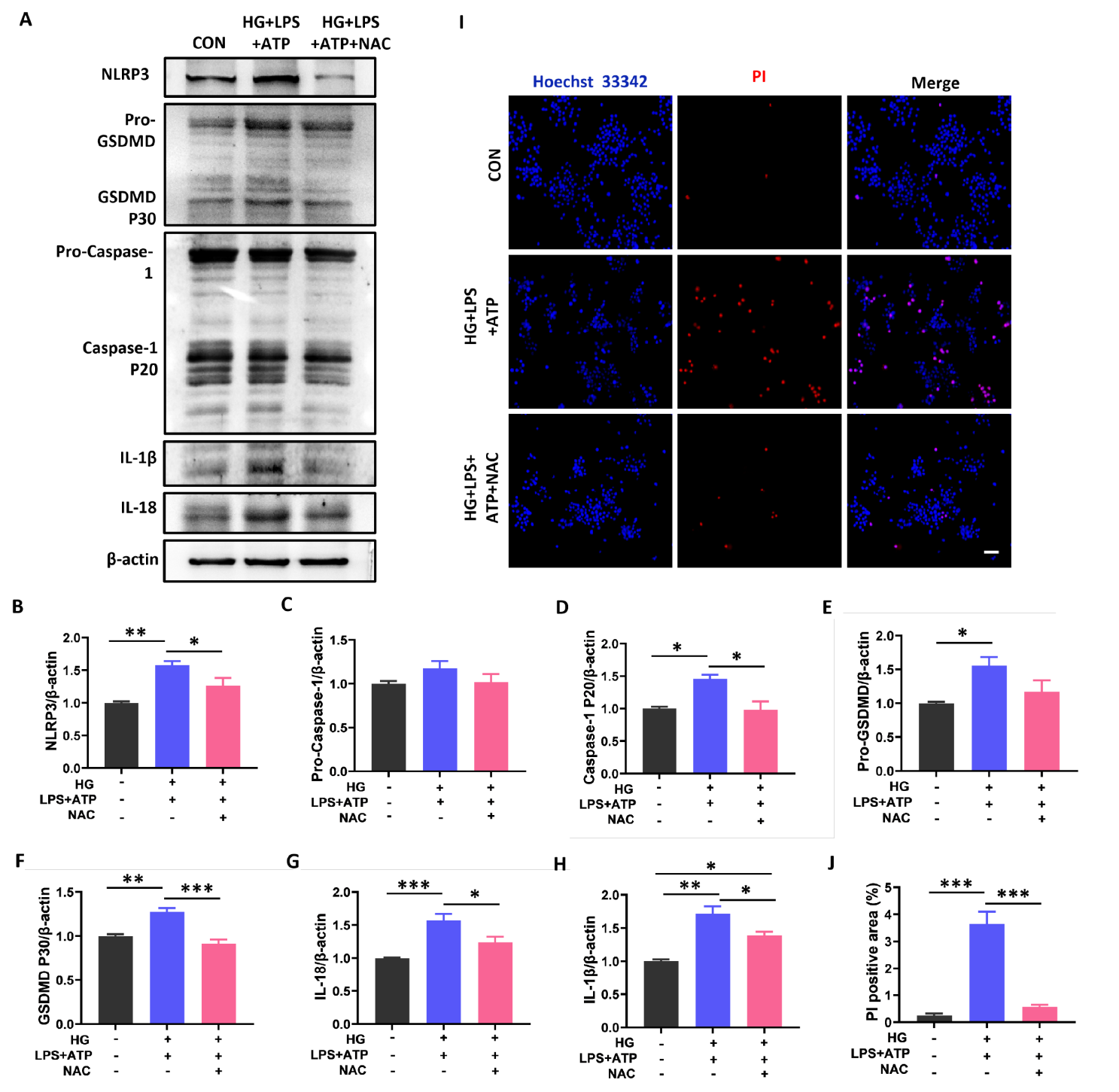
**

**Figure S2.** **NAC ameliorated HG-induced the excessive pyroptosis in BV-2 cells under LPS+ATP** **condition.** BV-2 cells were stimulated with HG (75 mM), LPS (1μg/ml), ATP (1 mM) and NAC (3 mM). The BV-2 cells were divided into three groups: CON, HG+LPS+ATP and HG+LPS+ATP+NAC. (A-H) WB results and statistical analysis of IL-18, Caspase-1, NLRP3, IL-1β and GSDMD in the BV-2 cells. (I and J) PI staining and statistical analysis of PI-positive signals of BV-2 cells, scale bar = 50 μm. n = 3, *p＜0.05, **p＜0.01, ***p＜0.001.

**Figure S3**

**
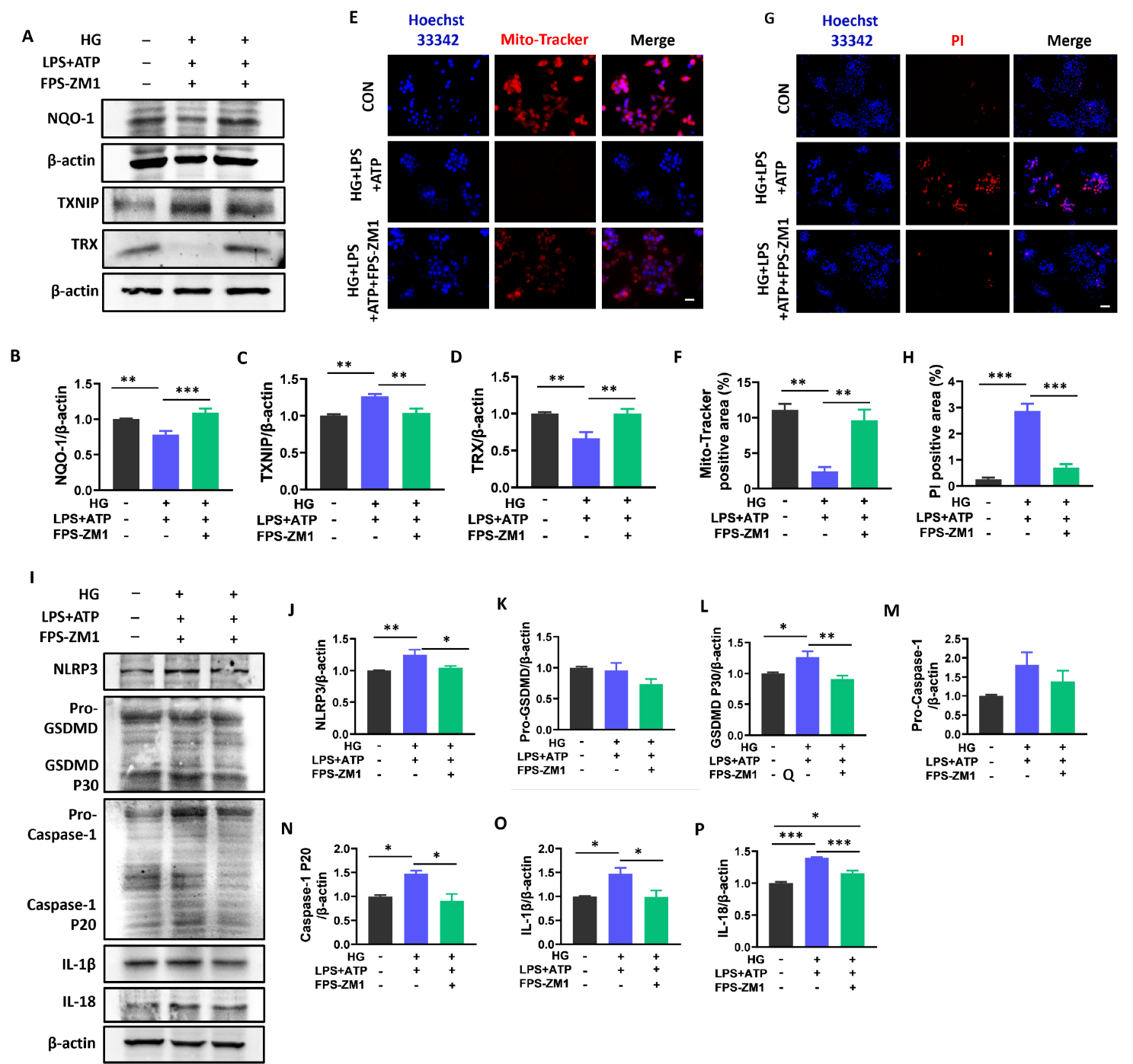
**

**Figure S3. FPS-ZM1 ameliorated HG-mediated the excessive oxidative stress and pyroptosis in BV-2 cells under LPS+ATP condition.** BV-2 cells were stimulated with HG (75 mM), LPS (1μg/ml), ATP (1 mM) and FPS-ZM1 (3 mM). The BV-2 cells were divided into three groups: CON, HG+LPS+ATP and HG+LPS+ATP+FPS-ZM1. (A-D) WB results and statistical analysis of NQO-1, TXNIP and TRX in the BV-2 cells. (E and F) The Mito-tracker staining of BV-2 cells and statistical analysis of Mito-tracker staining result. (G and H) The images and statistical analysis of PI-positive signals of BV-2 cells, scale bar = 50 μm. (I-P) WB results and statistical analysis of NLRP3, Caspase-1, IL-18, IL-1β and GSDMD in the BV-2 cells. n = 3, *p＜0.05, **p＜0.01, ***p＜0.001.
