# Supplementary figures and images for: Type 2 Diabetes Promotes the Microglial Pyroptosis by Activating NLRP3 Inflammasome to Impede Remyelination After Spinal Cord Injury
Source: Research (Wash D C). 2026 Apr 14;9:1237. doi: 10.34133/research.1237 (PMC13077133; doi:10.34133/research.1237)

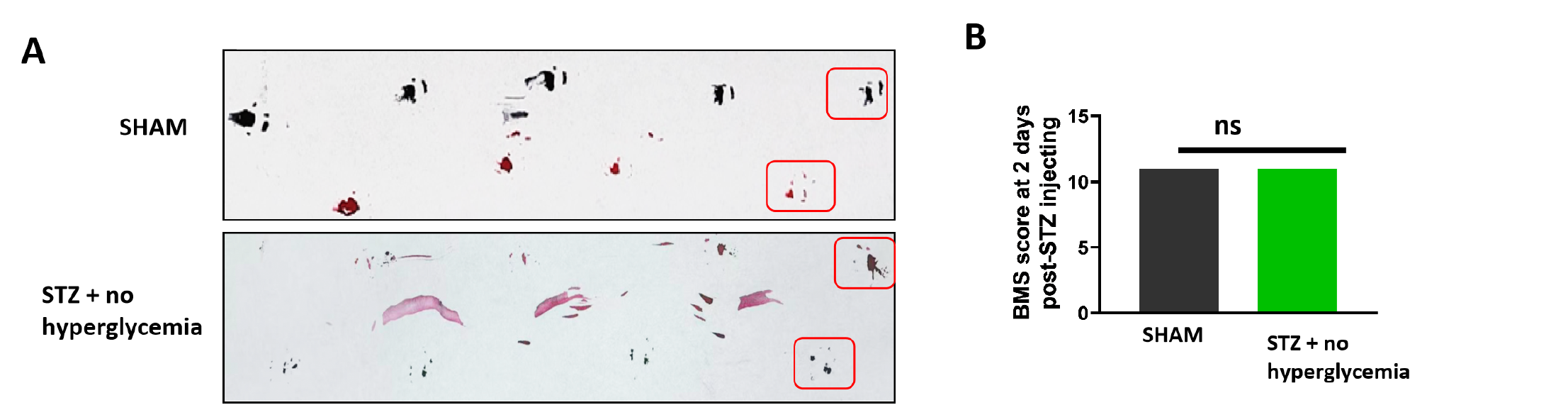

Supplement: Supplementary 1 — Figs. S1 to S3 [file research.1237.f1.zip › Supplementary Figure 1.tif]

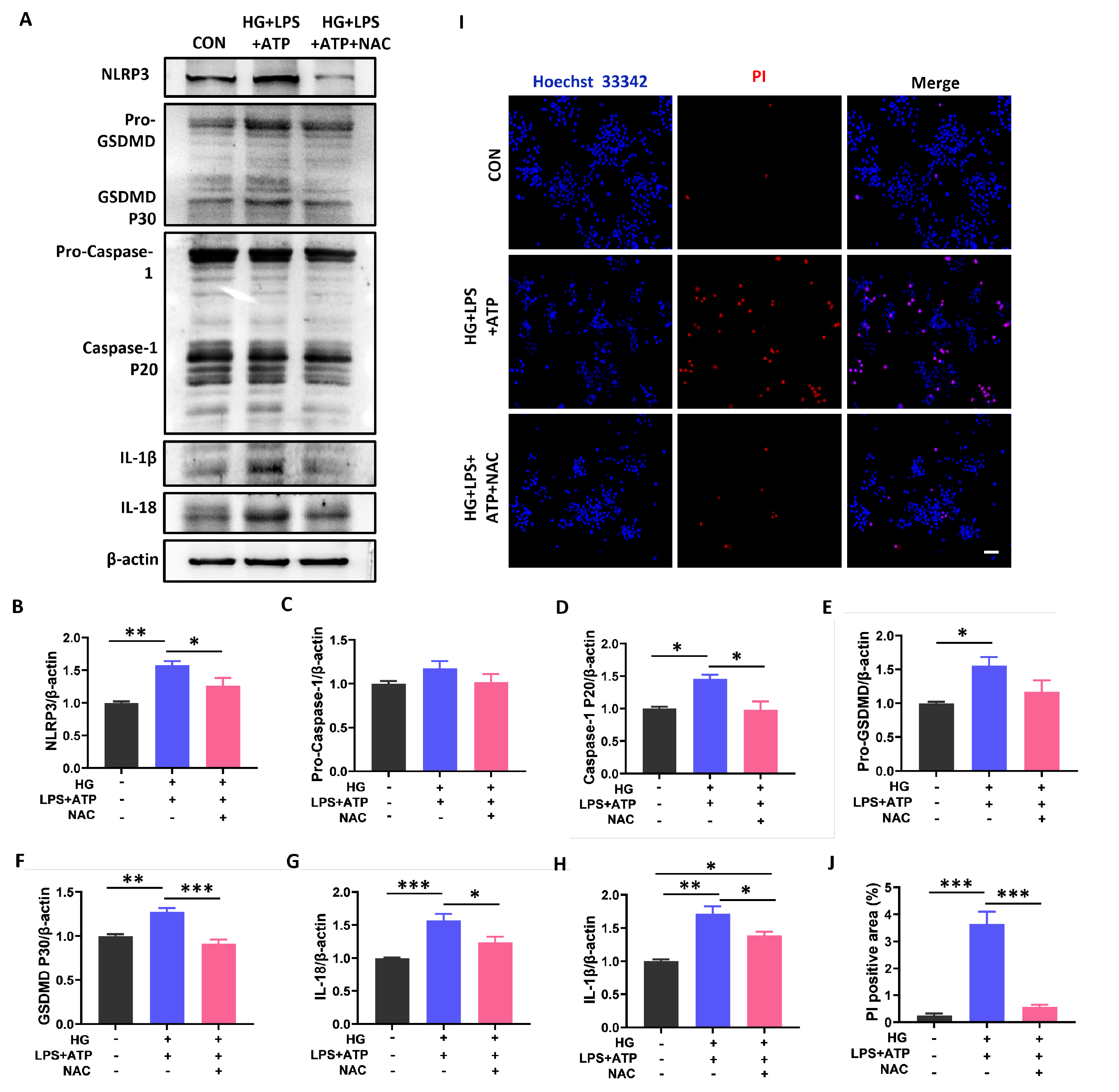

Supplement: Supplementary 1 — Figs. S1 to S3 [file research.1237.f1.zip › Supplementary Figure 2.tif]

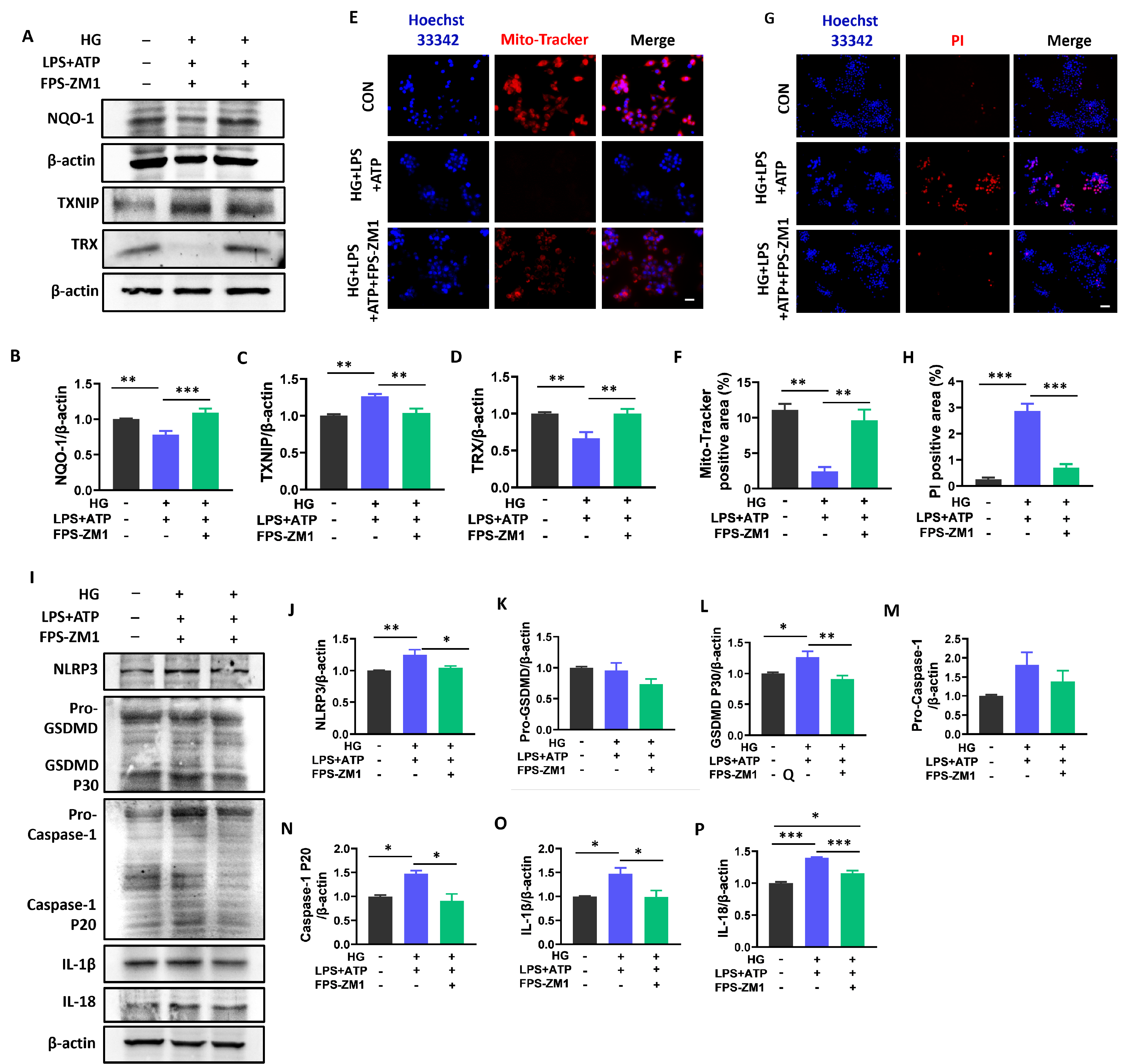

Supplement: Supplementary 1 — Figs. S1 to S3 [file research.1237.f1.zip › Supplementary Figure 3.tif]
